# Transcriptome Analysis of *Stephania tetrandra* and Characterization of Norcoclaurine-6-O-Methyltransferase Involved in Benzylisoquinoline Alkaloid Biosynthesis

**DOI:** 10.3389/fpls.2022.874583

**Published:** 2022-03-31

**Authors:** Kunlun Li, Xuefei Chen, Jianbo Zhang, Can Wang, Qiwei Xu, Jiangning Hu, Guoyin Kai, Yue Feng

**Affiliations:** ^1^Laboratory of Medicinal Plant Biotechnology, College of Pharmaceutical Sciences, The Third Affiliated Hospital, Zhejiang Chinese Medical University, Hangzhou, China; ^2^Zhejiang Conba Pharmaceutical Limited Company, Zhejiang Provincial Key Laboratory of Traditional Chinese Medicine Pharmaceutical Technology, Hangzhou, China

**Keywords:** *Stephania tetrandra*, biosynthetic pathways, transcriptome sequencing, (S)-norcoclaurine-6-O-methyltransferase, biochemical characterization

## Abstract

*Stephania tetrandra* (S. Moore) is a source of traditional Chinese medicine that is widely used to treat rheumatism, rheumatoid arthritis, edema, and hypertension. Benzylisoquinoline alkaloids (BIAs) are the main bioactive compounds. However, the current understanding of the biosynthesis of BIAs in *S. tetrandra* is poor. Metabolite and transcriptomic analyses of the stem, leaf, xylem, and epidermis of *S. tetrandra* were performed to identify candidate genes associated with BIAs biosynthesis. According to the metabolite analysis, the majority of the BIAs accumulated in the root, especially in the epidermis. Transcriptome sequencing revealed a total of 113,338 unigenes that were generated by *de novo* assembly. Among them, 79,638 unigenes were successfully annotated, and 42 candidate structural genes associated with 15 steps of BIA biosynthesis identified. Additionally, a new (S)-norcoclaurine-6-O-methyltransferase (6OMT) gene was identified in *S. tetrandra*, named St6OMT2. Recombinant St6OMT2 catalyzed (S)-norcoclaurine methylation to form (S)-coclaurine *in vitro*. Maximum activity of St6OMT2 was determined at 30°C and pH 6.0 in NaAc-HAc buffer. Its half-life at 50°C was 22 min with the *K_m_* and *k_cat_* of 28.2 μM and 1.5 s^−1^, respectively. Our results provide crucial transcriptome information for S. *tetrandra*, shedding light on the understanding of BIAs biosynthesis and further gene functional characterization.

## Introduction

*Stephania tetrandra* (S. Moore) is a traditional Chinese medicine, belonging to the subgenus *Botryodiscia* of the genus *Stephania* in the Menispermaceae family (Jiang et al., 2020; Zhang et al., 2020a). It has been widely applied to therapy of rheumatoid arthritis, rheumatism, edema, and hypertension (Jiang et al., 2020). Modern pharmacological studies have shown that it exhibits wide pharmacological activities, including anti-tumor, anti-inflammatory, neuroprotective, and antiviral effects (Semwal et al., 2010; Jiang et al., 2020; Zhang et al., 2020a). Previous chemical studies have documented that the active compounds of *S. tetrandra* include five types of benzylisoquinoline alkaloids (BIAs), including monobenzylisoquinolines, bisbenzylisoquinolines, aporphines, protoberberines, and tetrahydroprotoberberines (Jiang et al., 2020). Tetrandrine, a bisbenzylisoquinoline, is the main active compound (Bhagya and Chandrashekar, 2016). Several reports demonstrated that it has a notable effect on the fight against the Ebola virus (Sakurai et al., 2015) and COVID-19 (Heister and Poston, 2020; Ou et al., 2020). It is noted that commercial tetrandrine is mainly produced *via* extraction from traditional Chinese medicinal plants and chemical synthesis. However, limitations in medicinal plant resources and the complexity of chemical synthesis restrict its use, making its production insufficient in the face of growing demands. Therefore, it is necessary to develop new approaches to produce tetrandrine and BIAs.

The rapid development of metabolic engineering and synthetic biology has offered an alternative approach for the sustainable production of valuable natural products including morphine (Thodey et al., 2014), colchicine (Nett et al., 2020), and scopolamine (Srinivasan and Smolke, 2020). As an important plant secondary metabolite, tetrandrine biosynthesis is derived from L-tyrosine in plants. Previous studies have reported that the pathway from L-tyrosine to N-methylcoclaurine is similar to that of other BIAs (Bhagya and Chandrashekar, 2016; Zhang et al., 2020b). This biosynthetic pathway consists of several enzymes, including tyrosine decarboxylase, tyramine 3-hydroxylase, tyrosine aminotransferase, (S)-norcoclaurine synthase, (S)-norcoclaurine 6-O-methyltransferase (6OMT), and (S)-coclaurine N-methyltransferase. Cytochrome P450 enzymes and O-methyltransferases (OMTs) catalyze the conversion of N-methylcoclaurine to tetrandrine. However, the genes associated with the pathway of the tetrandrine biosynthesis of *S. tetrandra* remain unclear owing to insufficient genetic information. Furthermore, among these reported enzymes, 6OMT is a rate-limiting enzyme that catalyzes (S)-norcoclaurine to form (S)-coclaurine at its 6-hydroxyl group (Inui et al., 2007; Robin et al., 2016). Recently, 6OMTs isolated from *Thalictrum flavum* (Robin et al., 2016), *Coptis japonica* (Sato et al., 1994), *Nelumbo nucifera* (Menendez-Perdomo and Facchini, 2020), and *Papaver somniferum* (Ounaroon et al., 2003) were characterized, providing functional information on their catalysis. Accumulating reports suggest that 6OMT activity is correlated with the production of (S)-coclaurine and other BIAs, implying that regulation of the expression level of 6OMT might increase the production of BIAs. For example, higher levels of sanguinarine were detected in Cj6OMT-overexpressing *Eschscholzia californica* cells (Inui et al., 2007), whereas suppression of the transcript levels of 6OMT from *P. somniferum* significantly reduced total alkaloid accumulation (Desgagne-Penix and Facchini, 2012). Therefore, we speculated 6OMT might be an important enzyme in the production of BIAs in *S. tetrandra* as well.

Recently, transcriptome analysis and metabolic profiling have been extensively used to explore and identify critical genes related to the biosynthesis and regulation of secondary metabolites in medicinal plants, such as *Gloriosa superba* (Nett et al., 2020), *Cinnamomum camphora* (Chen et al., 2018), and *Atractylodes macrocephala* (Ruan et al., 2021). Using this integrative approach, several crucial genes ((S)-coclaurine N-methyltransferase, (S)-N-methylcoclaurine-3-hydroxylase) have been identified in *Corydalis yanhusuo* (Xu et al., 2021), *Coptis deltoidea* (Zhong et al., 2020), and *Erythrina velutina* Willd (Chacon et al., 2021). These results shed light on the biosynthesis of BIAs in *S. tetrandra*. Herein, an integrative approach of metabolic analysis and transcriptome sequencing was conducted to explore candidate genes related to tetrandrine biosynthesis and regulation. Under the annotated and classified of the functional genes, those genes associated with the biosynthesis and regulation of BIAs were identified. Moreover, a new 6OMT was isolated from *S. tetrandra* and then its enzymatic properties were assessed. These results shedding light on the underlying mechanism of BIAs biosynthesis and further gene functional characterization in *S. tetrandra*.

## Materials and Methods

### Plant Materials

Four-year-old *S. tetrandra* growing in the Medicinal Botanical Garden of Zhejiang Chinese Medical University were collected on August. Germplasm resource was provided by Zhejiang Conba Pharmaceutical Limited Company. Three biological replicate samples of stem, leaf, xylem, and epidermis were obtained by mixing equal amounts from every three of the nine plants, and then were immediately frozen in liquid nitrogen and stored at −80°C. Subsequently, the samples of each tissue were individually ground into powder and used for chemical composition analysis and RNA extraction.

### Alkaloid Extraction and Composition Analysis

Fifty milligram of the powders were mixed to 2 ml methanol and then sonicated extracts for 0.5 h. After centrifugation, the supernatants were filtered with a nylon syringe filter (0.22 μm). Quantitative analysis was conducted on an ultra-high-performance liquid chromatography-quadrupole time-of-flight mass spectrometry (UPLC-Q-TOF/MS) system (Waters, Milford, United States) equipped with a BEH C18 column (2.1*50 mm, 1.7 μm, Waters, Milford, United States). The mobile phase A and B were the 0.1% aqueous formic acid and methanol, respectively. The elution program was set as follow: 10% B at 0–1 min, 10–20% B at 1–5 min, 20–25% B at 5–15 min, 25–50% B at 15–25 min, 50–95% B at 25–30 min, and 95% B at 30–32 min. The column temperature and flow rate were 30°C and 0.25 ml·min^−1^, respectively. In the MS^E^ continuum model, an ESI^+^ mode was performed with the full scan monitoring in the range of m/z 50–1,200. The external standards of (S)-norcoclaurine, (S)-N-methylcoclaurine, and tetrandrine were purchased from Shanghai yuanye Bio-Technology Co., Ltd.; (S)-coclaurine was purchased from Chengdu purechem-standard Co., Ltd.; and fangchinoline was purchased from National Institutes for Food and Drug Control. And we perform a qualitative analysis of our test results with the UNIFI software (Waters, Milford, United States). A heatmap was generated using TBtools with row scaling to analyze the relative content of each BIAs. To assess the feature components of the detected components, a Principal Component Analysis (PCA) method was used in our study with Metware Cloud.^1^

### Transcriptome Sequencing and Functional Annotation

Total RNA from the stem, leaf, xylem, and epidermis of *S. tetrandra* was extracted using the RNA Prep Pure Plant kit (Tiangen Biotech, Beijing, China) according to the manufacturer’s protocol. The quality and quantity of RNA were measured using an Agilent 2100 Bioanalyzer (Agilent Technologies, United States), NanoDrop spectrophotometer (Thermo Fisher Scientific, United States), and agarose gel electrophoresis, respectively. RNA samples with OD260/280 ≥1.8 and the RNA Integrity Number ≥8.0 were sent to Novogene Biotech (Beijing, China) for next-generation sequencing using Illumina HiSeq. The low-quality sequences, adapter sequences, and unknown nucleotides were filtered from the raw reads collected from RNA-Seq. Then, the GC percent, Q20, and Q30 of each sample were calculated. Trinity software^2^ was applied to *de novo* assemble these clean reads, resulting in assembled transcripts and unigenes. After *de novo* assembly, seven functional databases were used to annotate the assembled unigenes. These databases include the NCBI non-redundant protein database and nucleotide database (NR and NT, respectively),^3^ Pfam,^4^ Swiss-Prot,^5^ the Gene Ontology database (GO),^6^ the euKaryotic Ortholog Groups (KOG),^7^ and the Kyoto Encyclopedia of Genes and Genomes (KEGG).^8^ The transcription factors (TFs) in *S. tetrandra* were predicted using the hmmscan tool of the iTAK software.^9^

### Gene Expression Levels Analysis

The expression level of unigenes was assessed by calculating their FPKM value. Differential expression analyses for each unigene in different tissues were performed using DESeq2 (Love et al., 2014). Herein, the differentially expressed genes (DEGs) were screened with the thresholds were a significance level of corrected value of p (padj) <0.05 and Log2 (fold change) >1. GOseq and KOBAS (2.0) software were applied for GO enrichment and KEGG pathway enrichment analyses, with the padj <0.05, which was considered significantly enriched among DEGs, respectively.

### Identification of Candidate Genes in the BIAs Pathway

To identify the candidate genes associated with the BIAs pathway of *S. tetrandra*, local tblastn 2.2.10 was performed against *S. tetrandra* unigene sequences using query sequences of BIA-producing plants obtained from the Swiss-Prot and GenBank databases (Supplementary Table S1). The resulting unigenes with high degrees of identity were selected as candidate genes. A heatmap was generated using TBtools with row scaling.

### Phylogenetic and Conserved Domain Analyses

On the basis of amino acid sequences of candidate genes and reference genes obtained from BIA-producing plants (Supplementary Table S1), phylogenetic analysis was performed using the MEGA11 software with the neighbor-joining tree algorithm. Bootstrap values generated after 1,000 copies are labeled on the branches. The conserved protein domain structures of candidate genes were analyzed on the ExPASy PROSITE^10^ and Batch CD-Search.

### Quantitative Real-Time Polymerase Chain Reaction Analysis

To evaluate the transcriptome data, quantitative real-time polymerase chain reaction (qRT-PCR) was conducted to determine the expression levels of 12 transcripts (including 10 functional genes and 2 TFs related to the BIA biosynthesis pathway) and using the *S. tetrandra* actin gene (Cluster-50127.14503) as a reference for normalization. qRT-PCR was conducted on an Applied Biosystems 7500 Real-Time PCR System (Thermo Fisher Scientific, Waltham, United States) using a PowerUp^™^ SYBR^™^ Green Master Mix (Thermo Fisher Scientific, Waltham, United States) as the manufacturer’s protocol. The primers listed in Supplementary Table S2. The 2^−ΔΔCT^ method was applied to calculate the relative expression levels of each transcripts. All assays were performed in triplicate.

### Construction of a Vector Containing Recombinant St6OMT2

The *St6OMT2* gene was cloned from the *S. tetrandra* transcriptome library using the 6OMT2-F and 6OMT2-R as primers (Supplementary Table S2). The Polymerase chain reaction (PCR) was performed using Prime STAR^®^ HS DNA Polymerase (TaKaRa, Dalian, China) in 50 μl of reaction mixture containing cDNA and the primers mentioned above. The PCR amplification conditions were as follows: 98°C for 3 min; 34 cycles of 98°C for 10 s, 60°C for 10 s, 72°C for 60 s; and 72°C extension for 10 min. The amplification products were recovered using the GeneJET Gel Extraction Kit (Thermo Fisher Scientific, Waltham, United States), then linked to the pET28a (Hua Yue Yang, Beijing, China) between the *BamH* I and *Hind* III restriction sites. The recombinant pET28a-St6OMT2 was sequenced by Sun Ya Biological (Hangzhou, China).

### Sequence Analysis of St6OMT2

The theoretical isoelectric point and molecular weight of St6OMT2 were predicted by The ExPASy Proteomics Server.^11^ The SignalP 4.1^12^ and TMHMM^13^ were conducted to analyze the possible signal peptide and transmembrane regions, respectively. Multiple sequence alignment between St6OMT2 and that of different species was performed using ClustalX2, and the other 6OMT sequences were downloaded from GenBank (Supplementary Table S1). Homology modeling of St6OMT2 was conducted on the SWISS-MODEL^14^ and the structure of *T. flavum* 6OMT (PDB: 5ICC) as the template (Robin et al., 2016). The graphical molecular representation was generated by the PyMOL.^15^

### Expression and Purification of Recombinant St6OMT2

Recombinant pET28a-St6OMT2 was transformed into *Escherichia coli* BL21 (DE3) for protein expression. Positive transformants were inoculated into 20 ml Luria-Bertani medium with kanamycin (100 μg·mL^−1^) at 37°C and 220 r·min^−1^ agitation for overnight culture. The seed culture was transferred into fresh Luria-Bertani medium and cultured under the same conditions for 2 h until the OD600 reached 0.5, and then adding 0.1 mM IPTG to induce the expression of recombinant St6OMT2 at 16°C for 24 h. After centrifugation, the recombinant strains were resuspended with phosphate buffer saline (PBS, pH 7.0). These strains were then disrupted by ultrasonication at 4°C for 5 min (250 W, 20 kHz, 2 s per time at 4 s interval) and the supernatant was collected under the same conditions.

To purify the recombinant St6OMT2, nickel affinity chromatography (GE Healthcare, Salt Lake City, United States) was conducted as our previous protocol (Feng et al., 2017). The fractions containing St6OMT2 were stored at 4°C. Proteins were analyzed by sodium dodecyl sulfate–polyacrylamide gel electrophoresis (SDS-PAGE). The Bradford method was conducted to determine the protein concentration with bovine serum albumin as the standard (Bradford, 1976).

### Enzymatic Activity of St6OMT2

St6OMT2 activity was detected by quantifying the liberation of (S)-coclaurine at 37°C for 30 min using a reaction mixture in 0.5 ml PBS, 100 μg purified St6OMT2, 1 mM (S)-norcoclaurine, and 1 mM SAM. An equivalent of heat-inactivated St6OMT2 was set as the negative control. The reaction was stopped by adding of 0.25 ml methanol. A rapid analysis method with a UPLC-TUV system (Waters, Milford, United States) was established to detect the reaction products. Equipped with the same BEH C18 column. The mobile phase A and B were the 0.03% trifluoroacetic acid and methanol, respectively. The elution program was set as follow: 18% B at 0–6 min and 18–95% B at 6–15 min. The column temperature and flow rate were 30°C and 0.25 ml·min^−1^, respectively. The reaction products were confirmed by UPLC-MS/MS (Waters, Milford, United States) based on the retention time and m/z of the external standard.

### Enzymatic Properties of St6OMT2

The optimum temperature for St6OMT2 catalysis was determined from 4 to 70°C. The thermal stability of St6OMT2 was examined by heat treatment at 50°C for 40 min and then determined their residual activity at 37°C. The optimum pH of the St6OMT2 catalysis was examined in 20 mM following buffer: NaAc-HAc buffer at pH 3.0 to 6.0, K_2_HPO_4_-KH_2_PO_4_ buffer at pH 6.0 to 8.0, and Tris–HCl buffer at pH 8.0 to 10.0. To explore the influence of metal ions (Na^+^, K^+^, Fe^2+^, Mn^2+^, Zn^2+^, Mg^2+^, Ca^2+^, Cu^2+^, Ba^2+^, and Co^2+^) and EDTA on St6OMT2 activity, enzyme solutions with 1 mM of each additive were incubated at 4°C for 24 h, and the residual activity was determined at 37°C. Kinetic constants were determined using (S)-norcoclaurine as the substrate with a concentration range of 10–200 μM at 37°C. *K_m_* and V_max_ were calculated by fitting the Hill function. The *k_cat_* was measured using the equation *k_cat_* = V_max_/[E], in which [E] is the content of St6OMT2. To measure its substrate specificity, the catalytic activity of St6OMT2 was detected using substrates with similar structures, including (S)-coclaurine, N-methylcoclaurine, and fangchinoline, under the same conditions.

## Results

### Determination of BIAs in *Stephania tetrandra*

**Figure 1:**
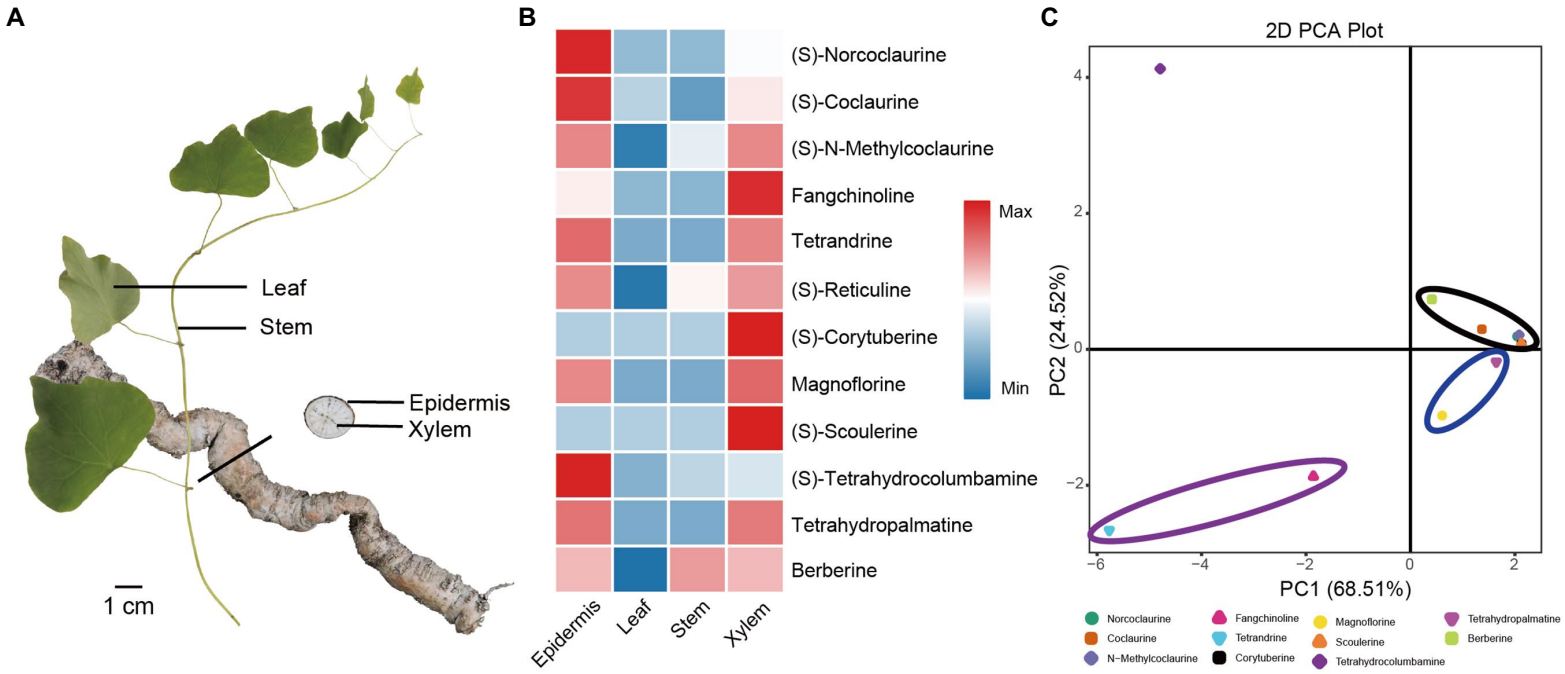
Quantitative analysis and PCA analysis of 12 BIAs in the stem, leaf, xylem, and epidermis of *Stephania tetrandra*. **(A)** Four tissues (stem, leaf, xylem, and epidermis) of *S. tetrandra* used for transcriptome sequencing and determination BIAs concentration. **(B)** Heat map of the relative content of 12 BIAs in each tissues. **(C)** PCA score plot of 12 BIAs.

BIAs are the major bioactive ingredients of *S. tetrandra* (Bhagya and Chandrashekar, 2016; Jiang et al., 2020). Among these BIAs, (S)-norcoclaurine, (S)-coclaurine, (S)-N-methylcoclaurine, and fangchinoline are important precursors of tetrandrine, which is the main medicinal component of *S. tetrandra*. Herein, UPLC-Q-TOF/MS was performed to quantify its relative concentrations in the stem, leaf, xylem, and epidermis (Figure 1A). As shown in Figure 1B and Supplementary Table S3, tetrandrine and its precursors showed a distinct tissue-specific distribution, and the majority of them accumulated in the root, especially in the epidermis. Specifically, the average contents of fangchinoline and tetrandrine were 8.37 and 17.31 mg·g^−1^ in epidermis, respectively (Supplementary Table S4). In addition, other BIAs were detected in this study. Compared with the important precursors of tetrandrine, magnoflorine, and (S)-tetrahydrocolumbamine were also mainly present in the epidermis. Furthermore, PCA results show that the different types of BIAs were clearly compartmentalized (Figure 1C). Tetrandrine can be used as the major BIAs Q-marker in *S. tetrandra* to distinguish epidermis from the other tissues.

### Transcriptome Sequencing and *de novo* Assembly of *Stephania tetrandra*

Twelve samples of stem, leaf, xylem, and epidermis of *S. tetrandra* were prepared and used for transcriptome sequencing. A total of about 723.6 million sequences of Illumina raw data were generated, leaving 698.5 million sequences after the original data was filtered with the GC percent of each sample was within 45–46% (Supplementary Table S5). After *de novo* assembly, 237,656 transcripts and 113,338 unigenes were obtained. The number of transcripts and unigenes larger than 1,000 bp was 44.42 and 24.15%, respectively. The N50 and average length of unigenes were 1,428 bp and 951 bp, respectively (Table 1).

**Table 1:** Summary of the sequence assembly.

Type	Transcript	Unigene
Total number	237,656	113,338
Total length (bp)	343,089,383	107,830,572
Maximum length (bp)	17,988	17,988
Minimum length (bp)	301	301
Mean length (bp)	1,444	951
N50 length (bp)	2,516	1,428
Length interval large than 1,000 bp	105,558	27,370

### Functional Annotation and Enrichment Analysis

As shown in Table 2, a total of 10,475 unigenes were annotated in all databases and 79,638 unigenes were annotated in at least one database. Specifically, a total of 51,024 unigenes in *S. tetrandra* were assigned to the NR database (Supplementary Figure S1). GO enrichment analysis revealed that 52,628 unigenes were annotated to the 3 major GO categories and 48 groups, namely, 24, 14, and 10 groups in “Biological Processes” category, “Cellular Components” category, and “Molecular Function” category, respectively (Supplementary Figure S2A). Through KOG analysis, these unigenes were classified into 25 groups and the “translation, ribosomal structure and biogenesis” group was the biggest (Supplementary Figure S2B). Based on KEGG analysis, a total of 23,965 unigenes were annotated to cellular processes, environmental information processing, genetic information processing, metabolism, and organismal systems (Supplementary Figure S2C). Among them, “Amino acid metabolism” (1946 unigenes) was the third representative pathways. Moreover, 513 unigenes were assigned to the subcategory “Biosynthesis of other secondary metabolites” in which 242 and 88 genes were assigned to the “Phenylpropanoid biosynthesis” (ko00940) and “Isoquinoline alkaloid biosynthesis” (ko00950), respectively (Supplementary Table S6).

**Table 2:** Statistics of annotations for assembled unigenes in public databases.

Database	Number of unigenes	Percentage (%)
Annotated in NR	51,024	45.01
Annotated in NT	51,179	45.15
Annotated in KEGG	23,965	21.14
Annotated in Swiss-Prot	52,473	46.29
Annotated in PFAM	52,628	46.43
Annotated in GO	52,628	46.43
Annotated in KOG	24,853	21.92
Annotated in all Databases	10,475	9.24
Annotated in at least one Database	79,638	70.26
Total Unigenes	113,338	100

### Differential Expression Analysis of Unigenes in *Stephania tetrandra*

**Figure 2:**
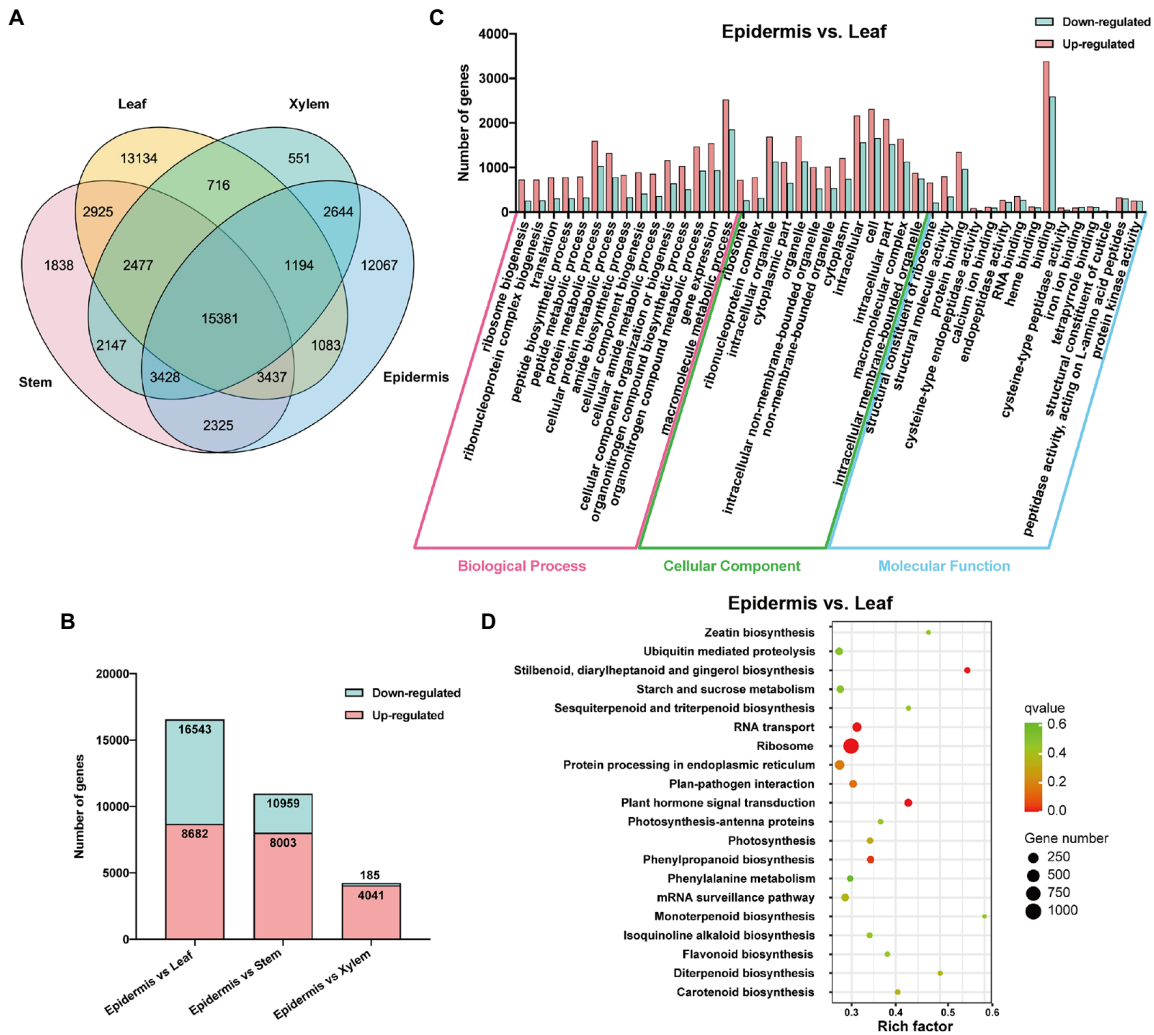
Functional annotation of DEGs. **(A)** The distribution of unigenes in different tissues. **(B)** Differentially expressed unigenes profiling of three libraries of epidermis vs. leaf, epidermis vs. stem, and epidermis vs. xylem of *Stephania tetrandra*. **(C)** GO enrichment analysis of DEGs in epidermis vs. leaf. **(D)** Scatterplot of KEGG pathway enrichment of DEGs in epidermis vs. leaf.

To investigate the distribution of assembled unigenes in each tissue, their relative expression levels were determined by calculating the fragment per kilobase of transcript per million fragments mapped (FPKM) values of assembled unigenes. As shown in Figure 2A, 15,381 unigenes were expressed in every tissues, whereas 15,262 unigenes were specifically expressed in the roots of *S. tetrandra*. Moreover, 2,644 unigenes were co-expressed in the xylem and epidermis. To explore the DEGs between the epidermis and other tissues of *S. tetrandra*, the expression levels of assembled unigenes were comparative analyzed. The distribution of DEGs in each group is shown in Figure 2B. Compared with the other two groups, epidermis and leaf had the largest number of DEGs (16,543 DEGs including 8,682 upregulated and 7,861 downregulated), indicating that there are many different-expressed unigenes between the epidermis and leaf of *S. tetrandra*. To further analyze the DEGs between epidermis and other three tissues, GO and KEGG enrichment analyses were conducted (Figure 2 and Supplementary Figure S3). According to the GO analysis, the DEGs related to “organonitrogen compound metabolic process” and “organonitrogen compound biosynthetic process,” were predominant in biological process category of epidermis and leaf (Figure 2C). Furthermore, KEGG analysis showed that 256 DEGs were assigned to secondary metabolite synthesis, and only a small proportion of DEGs were found in isoquinoline alkaloid biosynthesis in epidermis and leaf (Figure 2D). These DEGs upregulated in epidermis may be related to isoquinoline synthesis.

### Analysis of BIAs Biosynthesis Genes in *Stephania tetrandra*

**Figure 3:**
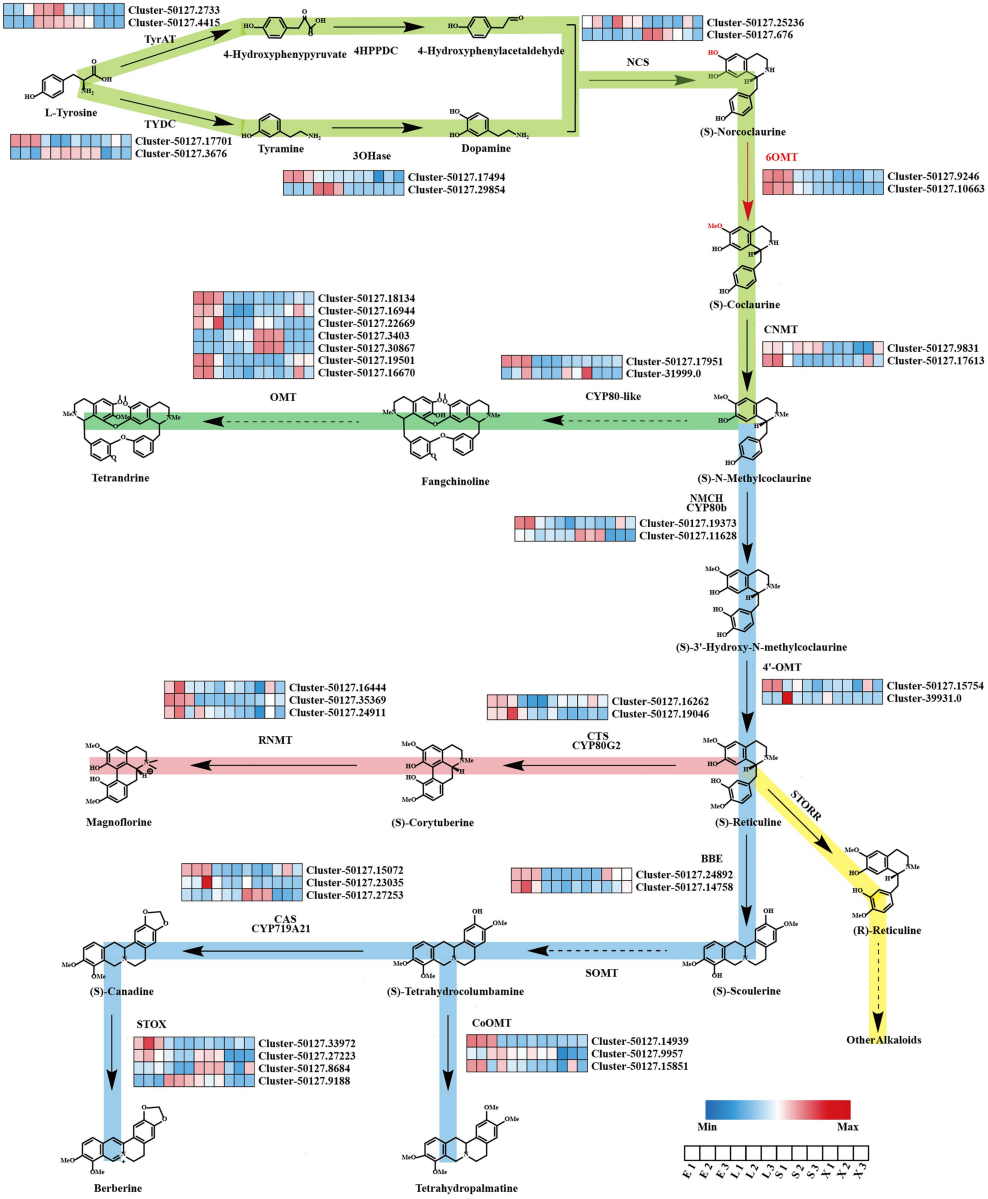
Putative biosynthesis pathways of BIAs and expression patterns of genes related to BIAs in *Stephania tetrandra*. E, epidermis; L, leaf; S, stem; X, xylem.

BIAs are the main active ingredients in the roots of *S. tetrandra*; however, the pathway in *S. tetrandra* has not yet been determined. The genes involve to the upstream pathway of BIAs biosynthesis, from tyrosine to N-methylcoclaurine, were predicted to be similar. We obtained candidate genes from *S. tetrandra* with high similarity to the BIA pathway genes of previously reported plants, namely, *P. somniferum*, *C. japonica*, and *E. californica*, by BLAST (Ounaroon et al., 2003; Inui et al., 2007; Ikezawa et al., 2008). As shown in Figure 3, a total of 12 unigenes encode tyrosine aminotransferase (2), tyrosine decarboxylase (2), tyramine 3-hydroxylase (2), (S)-norcoclaurine synthase (2), 6-OMT (2), and (S)-coclaurine N-methyltransferase (2). The results of phylogenetic analysis and domain analysis of candidate genes indicate that they had similar functions to the reference genes (Supplementary Figure S4). Moreover, to analyze their expression patterns, a heatmap was plotted based on the FPKM values of these genes in each tissue. Figure 3 revealed that the expression of these candidate genes was regulated in an organ-specific manner. Most of candidate genes were highly expressed in epidermis, which is consistent with BIAs accumulation. It is noted that the downstream biosynthetic pathway of tetrandrine from N-methylcoclaurine was unclear. Previous researches speculated that the CYP80 family and OMTs play indispensable roles in the downstream biosynthesis pathway of tetrandrine (Bhagya and Chandrashekar, 2016). In our dataset, we found that six unigenes belonged to the CYP80 family, whereas seven unknown unigenes encode OMTs. The results of the heatmap revealed that five OMTs were highly expressed in epidermis. These genes may be related to the biosynthesis of tetrandrine and need to be further explored in the future.

In addition, other BIAs, such as corytuberine, magnoflorine, tetrahydropalmatine, and berberine, might also be biosynthesized in *S. tetrandra* using N-methylcoclaurine as the fundamental precursor. As shown in Figure 3, 23 unigenes encode (S)-N-methylcoclaurine-3-hydroxylase (2), 3′-hydroxy-N-methyl-(S)-coclaurine 4-O-methyltransferase (2), berberine bridge enzyme (2), (S)-tetrahydroprotoberberine oxidase (4), CYP719 family (3), columbamine O-methyltransferase (3), (S)-corytuberine synthase (2), and reticuline N-methyltransferase (3). As shown in Supplementary Figure S4, most of the candidate proteins shared a similar evolutionary relationship and conserved domains with the known enzymes from other BIA-producing plants. Expression pattern analysis shows that the majority of candidate genes were highly expressed in the epidermis. These results suggest that these candidate genes may be responsible for the biosynthesis of BIAs in *S. tetrandra*.

### Classification and Differential Expression Analysis of Transcription Factor Family

**Figure 4:**
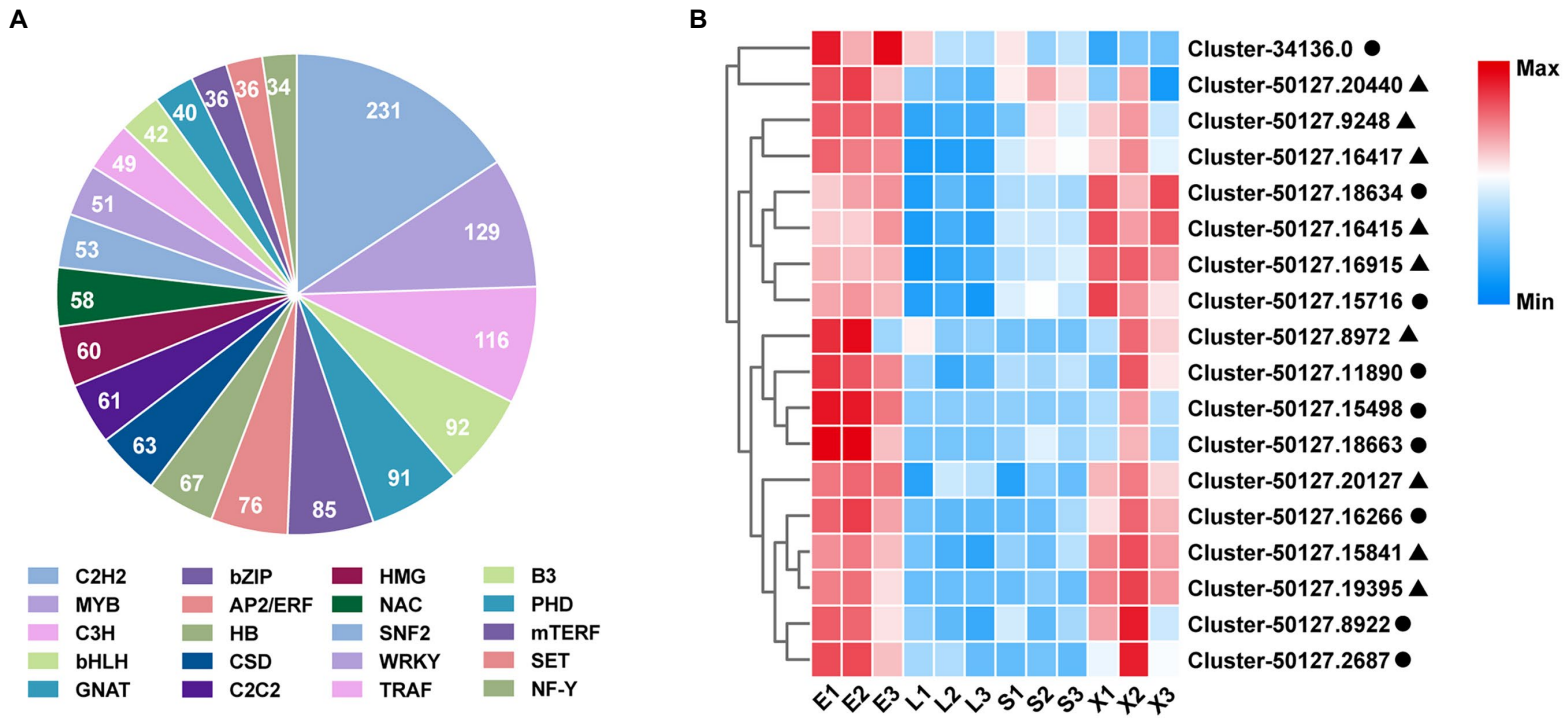
Classification of TF families **(A)** and expression analysis of bHLH and WRKY family **(B)**. Closed cycle and triangle represent bHLH and WRKY, respectively. E, epidermis; L, leaf; S, stem; X, xylem.

TFs are important regulatory proteins that play crucial roles in the regulation of plant growth (Pajerowska-Mukhtar et al., 2012), development (Xie et al., 2019), and secondary metabolism (Shi et al., 2021). A total of 2005 putative TFs in *S. tetrandra* were identified and matched to 72 TF families in this study. Among these families, the highest number of TFs were enriched in C2H2 (231 unigenes), followed by MYB (129 unigenes), C3H (116 unigenes), bHLH (92 unigenes), and GNAT (91 unigenes; Figure 4A). The bHLH and WRKY families have been reported to be the main TFs regulating BIA biosynthesis (Kato et al., 2007; Yamada et al., 2011, 2015). For example, the genes associated with berberine biosynthesis were significantly downregulated in CjbHLH1 and CjWRKY1-suppressing *C. japonica* cells (Kato et al., 2007; Yamada et al., 2011). In our transcriptomic data, nine bHLH and nine WRKY genes were highly expressed in the roots (Figure 4B). Phylogenetic analysis revealed that Cluster-34136.0 and Cluster-50127.19395 were closely homologous to EcbHLH1-1 and CjWRKY1, respectively (Supplementary Figure S5). These results indicate that both TFs might be associated with the regulation of BIAs biosynthesis in *S. tetrandra*. Further studies are necessary to ascertain the role and regulatory mechanisms of these TFs in subsequent research.

### Quantitative Real-Time Polymerase Chain Reaction Analysis of Candidate Genes in Each Tissues

To evaluate the transcriptome data, qRT-PCR analysis of 12 unigenes (10 functional genes and 2 TFs) was conducted. The results of qRT-PCR analysis revealed that the expression patterns of selected unigenes were similar with the RNA-Seq data (Supplementary Figure S6). Correlation analysis revealed that the correlation coefficients of epidermis vs. leaf, epidermis vs. stem, and epidermis vs. xylem were 0.84, 0.82, and 0.91, respectively (Supplementary Figure S7). These results validate the reliability of transcriptome data estimated using RNA-Seq.

### Identification and Functional Verification of St6OMT2

**Figure 5:**
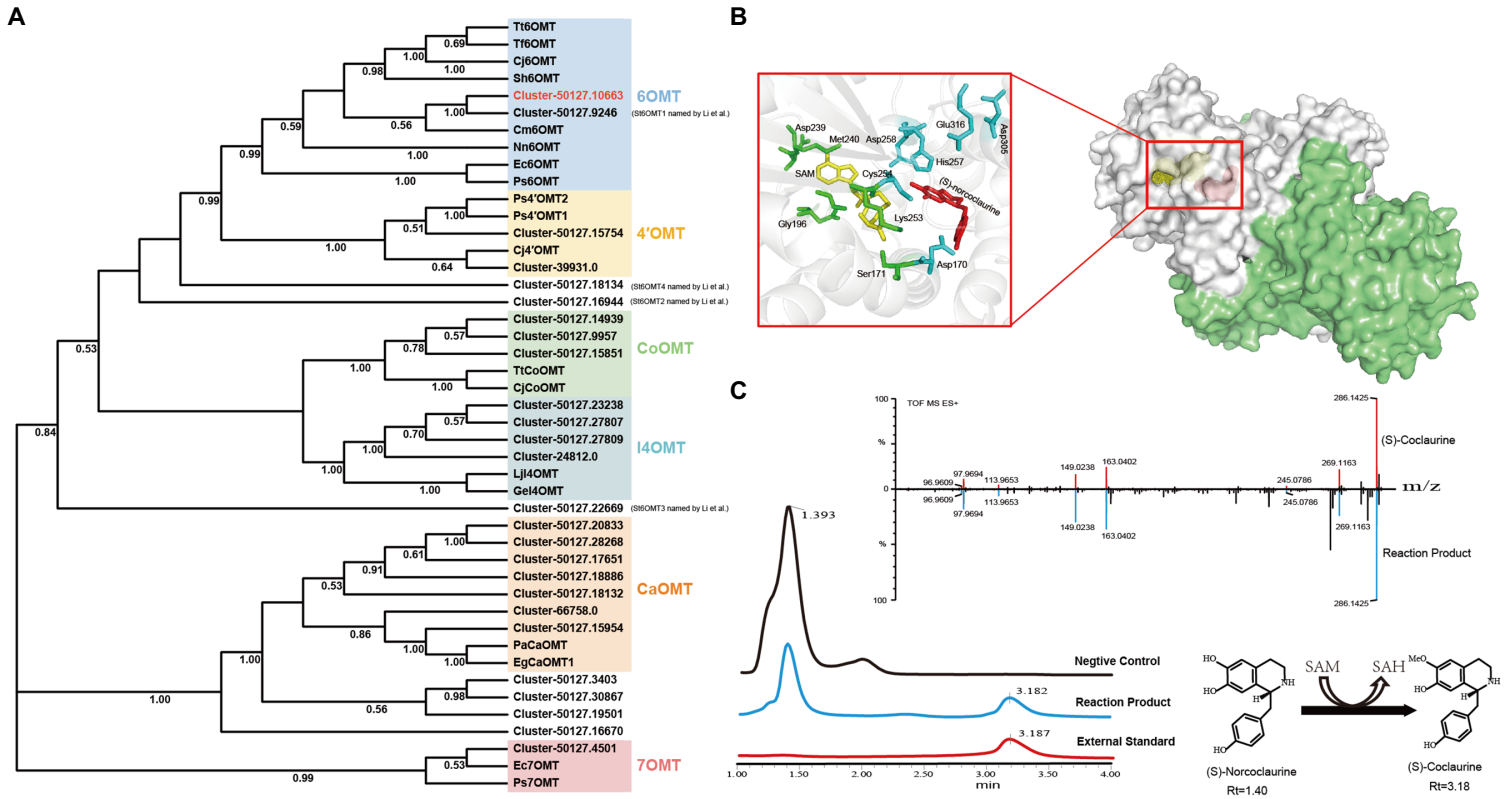
Phylogenetic relationships of OMTs and catalytic activity analysis recombinant St6OMT2. **(A)** Phylogenetic tree of OMTs. 6OMT: (S)-norcoclaurine-6-O-methyltransferase, 4′OMT: 3′-hydroxy-N-methyl-(S)-coclaurine 4-O-methyltransferase, CoOMT: columbamine O-methyltransferase, CaOMT: caffeic acid O-methyltransferase, I4OMT: isoflavone 4-O-methyltransferase, 7OMT: reticuline 7-O-methyltransferase. **(B)** Structural analysis of recombinant St6OMT2. Overall view of the St6OMT2 dimer structure and shown in surface representation. The ligand (S)-norcoclaurine and SAM were displayed in dots and colored yellow and red, respectively. In the close-up view, the substrate pocket is shown in cartoon representation, and the residues binding to SAM and (S)-norcoclaurine were displayed in stick and colored green and cyan. The ligand (S)-norcoclaurine and SAM were displayed in stick and colored red and yellow, respectively. **(C)** Enzymatic activity of recombinant St6OMT2 on (S)-norcoclaurine assayed by UPLC.

OMTs, such as 6OMT, 3′-hydroxy-N-methyl-(S)-coclaurine 4-O-methyltransferase, and columbamine O-methyltransferase, contribute to the formation of several secondary metabolites, including alkaloids. Herein, the majority of candidate OMTs clustered into six major clades (Figure 5A). It has been reported that 6OMT catalyze the rate-limiting step in the production of BIAs (Inui et al., 2007; Robin et al., 2016). Two 6OMTs (cluster-50127.10663, cluster-50127.9246) were annotated in our transcriptome, of which the cluster-50127.9246 was consistent with the St6OMT1 reported by Li et al. (2020). To explore the function of cluster-50127.10663, we amplified it from the *S. tetrandra* transcriptome library and named it St6OMT2. The obtained sequence of St6OMT2 was 1,053 bp and encoded 350 amino acids, with the expected molecular weight and isoelectronic point of 40.6 kDa and 5.91, respectively. Based on transmembrane region and signal peptide analyses, no transmembrane region or signal peptide was found in St6OMT2. Multiple sequence alignment showed that the active site and SAM attachment site of St6OMT2 were highly conserved with those of 6OMT from other plants. Specifically, the putative St6OMT2 active site (Asp170, Cys254, His 257, Asp258, Asp307, and Glu316) and SAM attachment site (Ser171, Gly196, Asp219, Asp239, Met240, and Lys253) were structurally equivalent to those in *T. flavum* 6OMT active site (Asp169, Cys253, His256, Asp257, Asp306, and Glu315) and SAM site (Thr170, Gly195, Asp218, Asp238, Met239, and Lys252; Supplementary Figure S8). Structural simulations show that this protein had a similar substrate binding pattern to 6OMT derived from *T. flavum* (67.25%), where six conserved residues were surrounded by (S)-norcoclaurine (Figure 5B). These data suggested that it was a potential 6OMT belonging to the SAM-dependent OMT family.

To explore the catalytic activity of St6OMT2, this enzyme was recombinantly expressed in *E. coli* BL21 (DE3) and purified to homogeneity. SDS-PAGE analysis revealed that the recombinant St6OMT2 was soluble in the intracellular supernatant and its molecular weight was 40 kDa, which agreed with the predicted value (Supplementary Figure S9). To analyze the catalytic activity of recombinant St6OMT2, the S-adenosyl-L-methionine and (S)-norcoclaurine were used as the methyl donor and substrate, respectively. Compared with the negative control group, a peak of 3.18 min was detected in the reaction mixture of St6OMT2. This was in agreement with the standard of (S)-coclaurine. UPLC-QTOF-MS/MS analysis confirmed that the production was (S)-coclaurine (Figure 5C). These results indicate that St6OMT2 had the ability to catalyze (S)-norcoclaurine methylation to form (S)-coclaurine.

### Characterization of Recombinant St6OMT2

**Figure 6:**
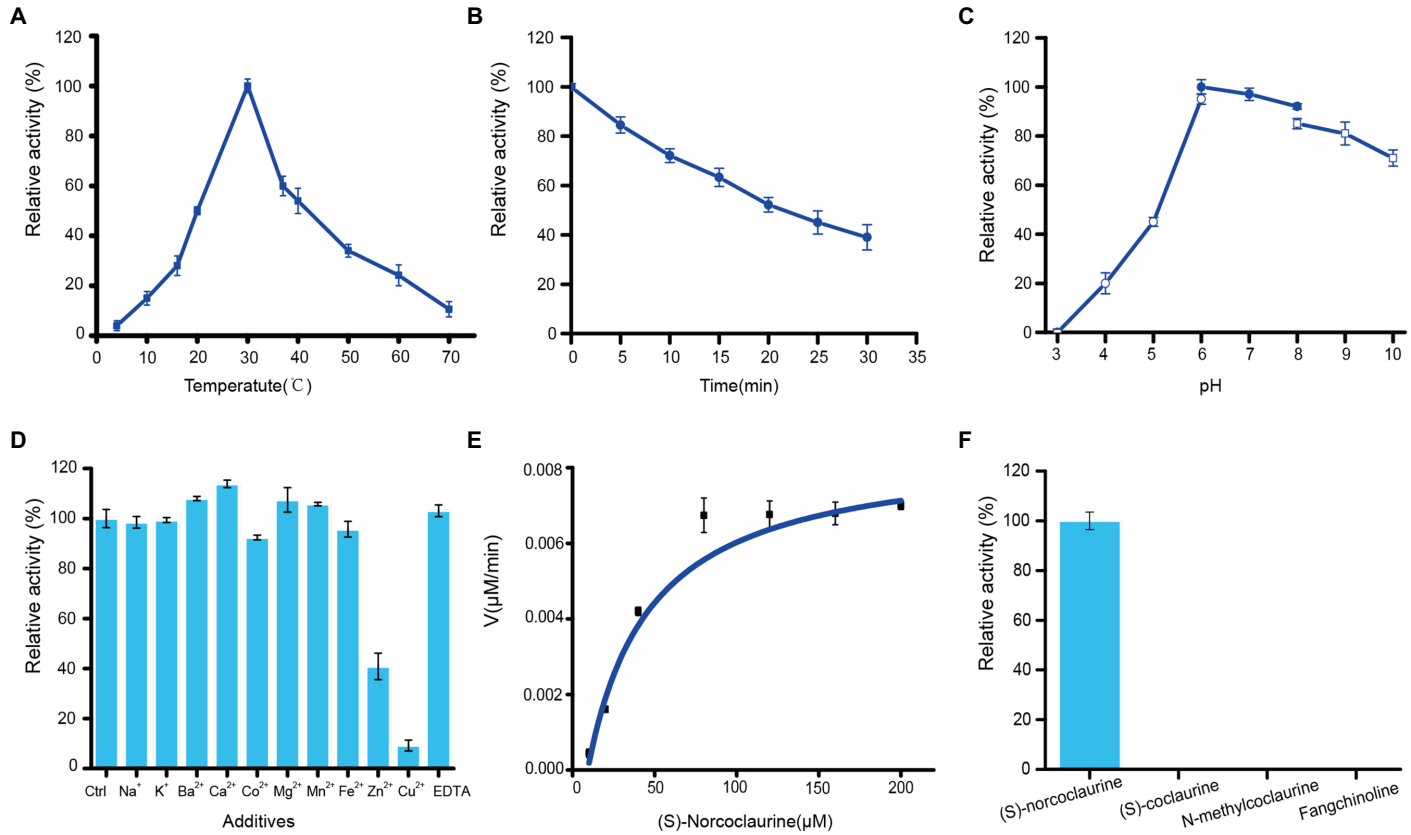
Enzymatic properties of recombinant St6OMT2. **(A)** Influence of temperature on the St6OMT2 activity. **(B)** Influence of thermostability of the St6OMT2 at 50°C. **(C)** Influence of pH on the St6OMT2 activity. **(D)** Influence of EDTA and metal ions on St6OMT2 activity. **(E)** Enzyme kinetics of St6OMT2 calculated by fitting with the Hill function. **(F)** Substrate specificity of St6OMT2 toward (S)-norcoclaurine, (S)-coclaurine, N-methyltransferase, and fangchinoline.

The effect of temperature on St6OMT2 activity is displayed in Figure 6A, in which the optimization temperature of St6OMT2 was 30°C and retained 15, 20% activity at 10°C and 70°C, respectively. Thermostability analysis showed that the half-life was 22 min at 50°C (Figure 6B). Figure 6C reveals the influence of pH on St6OMT2 activity, in which the optimization pH of St6OMT2 was 6.0 (NaAc-HAc buffer) and obtained high catalytic activity from 6.0 to 9.0. Figure 6D shows the influence of metal ions and EDTA on St6OMT2 activity, in which most of the metal ions showed a negligible effect on St6OMT2 activity, except for Zn^2+^ and Cu^2+^. Kinetic parameters were measured using (S)-norcoclaurine as the substrate, with *K_m_* and *k_cat_* values being 28.2 μM and 1.5 s^−1^, respectively (Figure 6E). The substrate specificity of St6OMT2 was detected by using the S-adenosyl-L-methionine as the methyl donor and (S)-coclaurine, N-methylcoclaurine, and fangchinoline as the substrates at the same conditions. As shown in Figure 6F, (S)-norcoclaurine was a preferential substrate than (S)-coclaurine, N-methylcoclaurine, and fangchinoline. These results also suggest that St6OMT2 might only catalyze the methylation of the 6-hydroxyl group.

## Discussion

Tetrandrine has attracted attention owing to its ability to fight against Ebola virus (Sakurai et al., 2015) and COVID-19 (Ou et al., 2020). As a traditional Chinese medicine rich in tetrandrine, the biosynthesis pathways of tetrandrine in *S. tetrandra* have drawn the attention of many researchers. However, the molecular mechanisms of tetrandrine biosynthesis and regulation in *S. tetrandra* remain unclear. In recent years, the development of transcriptome and metabolome approaches has provided guidance for the rapid identification of candidate genes associated with secondary metabolites biosynthesis in plants. For instance, Zhou et al. (2021) annotated the biosynthesis pathways of terpenoids, flavonoids, and phenylpropanoids by performing metabolic and transcriptome profiling of *Perilla frutescens*. Similarly, Li et al. (2021) identified a terpenoid synthase with the function of synthesizing terpenoids from *Colquhounia coccinea* var. mollis by the combined utilization of transcriptome and metabolite analyses. A similar integrative approach was performed in the present study; as a result, 12 chemical components were detected, and a new St6OMT2 was identified to be involved in BIAs biosynthesis. It is worth noting that there are distinct differences in the content of medicinally active components and the expression of related pathway genes in the epidermis, rhizome, xylem, and other underground tissues of root medicinal plants, such as *Salvia miltiorrhiza* (Xu et al., 2015, 2016), *Lithospermum erythrorhizon* (Takanashi et al., 2019), and *A. macrocephala* (Ruan et al., 2021). This indicates that the analysis of different tissues may provide a more accurate reference for functional gene identification in secondary metabolism biosynthesis. Nett et al. (2020) elucidated the pathway of colchicine biosynthesis by precisely analyzing colchicine content and transcriptomes in rhizomes, roots, stems, and leaves of *G. superba*. It has been reported that the epidermis is an important tissue for secondary metabolite biosynthesis. Based on metabolite analysis, we found that most of the precursors of tetrandrine were found in the epidermis, which suggests that tetrandrine is mainly biosynthesized in the epidermis of *S. tetrandra*.

A total of 79,638 unigenes were annotated and 15 steps involved in the BIAs pathway were identified in the present study. Moreover, 42 candidate genes related to the BIAs pathway obtained herein might be give us a precise scope for identified the unknown pathway in *S. tetandra*. These results might be providing more information than previous reports in which 31,994 unigenes were annotated 7 steps in the BIAs pathway were obtained (Zhang et al., 2020b). Previous research speculated that CYP450s might be related to its downstream biosynthesis (Zhang et al., 2020b). Specifically, CYP80A, CYP80B, and CYP80G catalyze C-O phenol coupling, hydroxylation, and C-C phenol coupling, respectively, whereas CYP719A catalyzes methylenedioxy bridge formation (Dastmalchi et al., 2018; He et al., 2018). Nine DEGs encoding these CYP450s were found in our transcriptome. Expression pattern analysis shows that five candidate CYP450s were highly expressed in the epidermis, which was agreed with the accumulation of tetrandrine. These results indicated that these five candidate CYP450s might be responsible for BIAs biosynthesis. Gene cloning and functional characterization of these candidate CYP450s will be performed in our subsequent study. Compared with the BIAs production plants, such as *Coptis chinensis* (He et al., 2018) and *Corydalis yanhusuo* (Xu et al., 2021), tetrandrine was the unique compound of *S. tetandra*. However, the gene related to this biosynthesis pathway was unknown. It was supposed that CYP80A family could catalyze C-O phenol coupling reaction (Kraus and Kutchan, 1995; Ikezawa et al., 2008). A specifically expressed CYP80 was identified in our transcriptome, we will do further study to elucidate its function. Taken together, the transcriptome resource obtained herein is highly valuable for shedding light on the tetrandrine and other BIA biosynthetic pathways.

In addition, OMTs were the crucial enzymes in the O-methylation process of the biosynthesis pathways of secondary metabolites. For example, 10-hydroxycamptothecin O-methyltransferase is an important protein in the 10-methoxycamptothecin biosynthesis pathway in *Camptotheca acuminate* (Salim et al., 2018), whereas SbOMT3 specifically transfers a methyl group to the 7-OH of flavonoids in *Scutellaria baicalensis* Georgi (Cui et al., 2022). Moreover, previous studies have revealed that several OMTs are also involved in the biosynthesis of BIAs. For example, TfS9OMT (Scoulerine 9-O-methyltransferase) catalyzes the conversion of (S)-scoulerine to (S)-tetrahydrocolumbamine in *T. flavum* (Valentic et al., 2020). The *Cj4*’OMT of *C. japonica* catalyzes the conversion of (S)-3′-hydroxy-N-methylcoclaurine to (S)-reticuline (Morishige et al., 2000). In the present study, we discovered two, two, and three unigenes encoding 6OMT, 3′-hydroxy-N-methyl-(S)-coclaurine 4-O-methyltransferase, and columbamine O-methyltransferase, respectively. We speculated these genes might be responsible for the biosynthesis of BIAs. These results will accelerate synthetic biology studies of BIAs. In addition, previous studies have speculated that OMTs might be involved in the O-methylation of fangchinoline to tetrandrine (Bhagya and Chandrashekar, 2016; Zhang et al., 2020b). However, the genes involved in this catalytic reaction are unclear. Seven unknown OMTs that were highly expressed in roots were also found in our study, and we speculate that these OMTs might be involved in the catalytic processes. Therefore, these genes need to be further studied.

It has been reported that 6OMT is a rate-limiting enzyme in the production of BIAs. Inui et al. (2007) found that the production of sanguinarine was significantly improved in Cj6OMT-overexpressing *E. californica* cells. Similarly, the suppression of the transcript levels of 6OMT from *P. somniferum* significantly reduced total alkaloid accumulation (Desgagne-Penix and Facchini, 2012). In this study, we annotated a new 6OMT from the transcriptome and named it St6OMT2. It was different with the St6OMTs reported by Li et al. (2020). Phylogenetic tree analysis showed that it was homologous with Cm6OMT (69%), whereas multiple sequence alignment revealed that the active site and SAM attachment site were structurally equivalent to those of *T. flavum* (Robin et al., 2016). Moreover, we have demonstrated that it can catalyze the (S)-norcoclaurine to form (S)-coclaurine *in vitro*. Compared with *St*6OMT1 reported by Li et al. (2020) *St*6OMT2 shared a similar expression pattern. Both enzymes were specifically expressed in the roots, which is in agreement with the fact that BIAs were accumulated in the roots of *S. tetrandra*. Additionally, as shown in Table S5, the catalytic activity of St6OMT2 matched that of St6OMT1 identified by Li et al. (2020). These results suggested that both enzymes might play a similar role in the biosynthesis of BIAs in *S. tetrandra*.

The catalytic properties of the different 6OMTs are listed in Supplementary Table S7. The optimum temperature and pH of St6OMT2 were 30°C and 6.0, respectively, which are close to those of *N. nucifera* (Menendez-Perdomo and Facchini, 2020). In addition, compared with the St6OMT1 reported by Li et al. (2020) St6OMT2 shared a similar catalytic property, that of only methylating the hydroxyl of (S)-norcoclaurine at the C6 position. This result contrasts with that of *Coptis chinensis* (He et al., 2018) and *Glaucium flavum* (Chang et al., 2015), in which they not only methylate the hydroxyl of (S)-norcoclaurine at the C6 position but also methylate the hydroxyl of other sites to form by-products. Moreover, many substrates including norlaudanosine can cause methylation by several 6OMTs as well (Frick and Kutchan, 1999; Frick et al., 2001; Ounaroon et al., 2003). The recombinant St6OMT2 obtained herein only methylates the (S)-norcoclaurine at the C6 position, whereas no peak was detected in other substrates, including (S)-coclaurine and fangchinoline. These results indicate that this enzyme might be not methylate the hydroxyl of other sites except for the C6 position. Therefore, we provide a promising 6OMT for the synthetic biology of BIAs owing to its advantage in reducing the formation of by-products.

In conclusion, an integrative approach of metabolic analysis and transcriptome sequencing was conducted to identify candidate genes responsible for tetrandrine biosynthesis and regulation in *S. tetrandra*. Metabolite analysis revealed that most of the precursors of tetrandrine were found in roots, especially in the epidermis. According to the transcriptome profiles, most genes annotated to the BIAs biosynthesis pathway displayed higher expression in the epidermis. We conclude that these genes may be responsible for the biosynthesis and regulation of BIAs. Moreover, a new 6OMT was identified from transcriptome data of *S. tetrandra*. The optimum temperature and pH of the recombine St6OMT2 were 30°C and 6.0, respectively. Taken together, our work provides valuable genetic information on *S. tetrandra* and sheds light on the biosynthesis of BIAs in this medicinal plant.

## Data Availability Statement

The datasets presented in this study can be found in online repositories. The names of the repository/repositories and accession number(s) can be found at: https://ngdc.cncb.ac.cn/gsa/, CRA005922.

## Author Contributions

YF, GK, KL, and XC conceived the project. KL, XC, and JZ conducted the experiments. CW, QX, and JH participated in the data analysis. YF, KL, and XC wrote the manuscript. All authors contributed to the article and approved the submitted version.

## Funding

This work was supported by National Natural Science Fund of China (82104337 and 82073963); Zhejiang Provincial Natural Science Foundation of China (LQ20C010004); the Major Science and Technology Projects of Breeding New Varieties of Agriculture in Zhejiang Province (2021C02074); Zhejiang Provincial Ten Thousand Program for Leading Talents of Science and Technology Innovation (2018R52050); Zhejiang Provincial Program for the Cultivation of High-Level Innovative Health Talents; the Research Project of Zhejiang Chinese Medical University (2021JKZKTS019B, 2021JKZKTS022B, and 2021JKZDZC06); and Opening Project of Zhejiang Provincial Preponderant and Characteristic Subject of Key University (Traditional Chinese Pharmacology) of Zhejiang Chinese Medical University (ZYAOX2018003 and ZYAOXYB2019009).

## Conflict of Interest

JH was employed by Zhejiang Conba Pharmaceutical Limited Company.

The remaining authors declare that the research was conducted in the absence of any commercial or financial relationships that could be construed as a potential conflict of interest.

## Publisher’s Note

All claims expressed in this article are solely those of the authors and do not necessarily represent those of their affiliated organizations, or those of the publisher, the editors and the reviewers. Any product that may be evaluated in this article, or claim that may be made by its manufacturer, is not guaranteed or endorsed by the publisher.

## Footnotes

^1^ https://www.metware.com

^2^ https://github.com/trinityrnaseq/trinityrnaseq

^3^ ftp://ftp.ncbi.nlm.nih.gov/blast/db/

^4^ http://pfam.xfam.org/

^5^ http://www.ebi.ac.uk/uniprot/

^6^ http://www.geneontology.org

^7^ http://www.ncbi.nlm.nih.gov/COG/

^8^ http://www.genome.jp/kegg

^9^ http://itak.feilab.net/cgi-bin/itak/index.cgi

^10^ http://www.expasy.ch/prosite/

^11^ https://web.expasy.org/protparam/

^12^ http://www.cbs.dtu.dk/services/SignalP/

^13^ http://www.cbs.dtu.dk/services/TMHMM/

^14^ http://swissmodel.expasy.org

^15^ http://www.pymol.org
