# Supplementary material for: Transcriptome Analysis of Stephania tetrandra and Characterization of Norcoclaurine-6-O-Methyltransferase Involved in Benzylisoquinoline Alkaloid Biosynthesis
Source: Front Plant Sci. 2022 Mar 31;13:874583. doi: 10.3389/fpls.2022.874583 (PMC9009073; doi:10.3389/fpls.2022.874583)
Supplement: Supplementary file 1 [file Data_Sheet_1.docx]

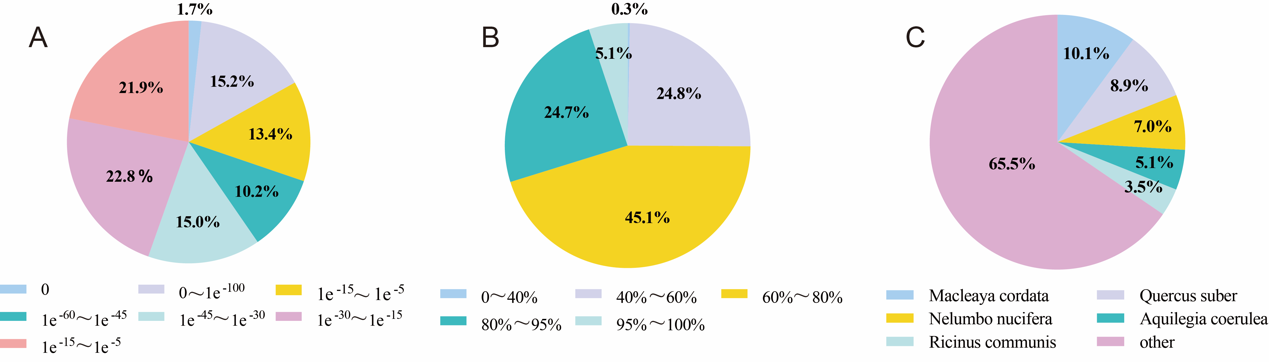


**Supplementary Figure S1** E-value (A), similarity (B), and species distribution (C) of the NR annotation.

**
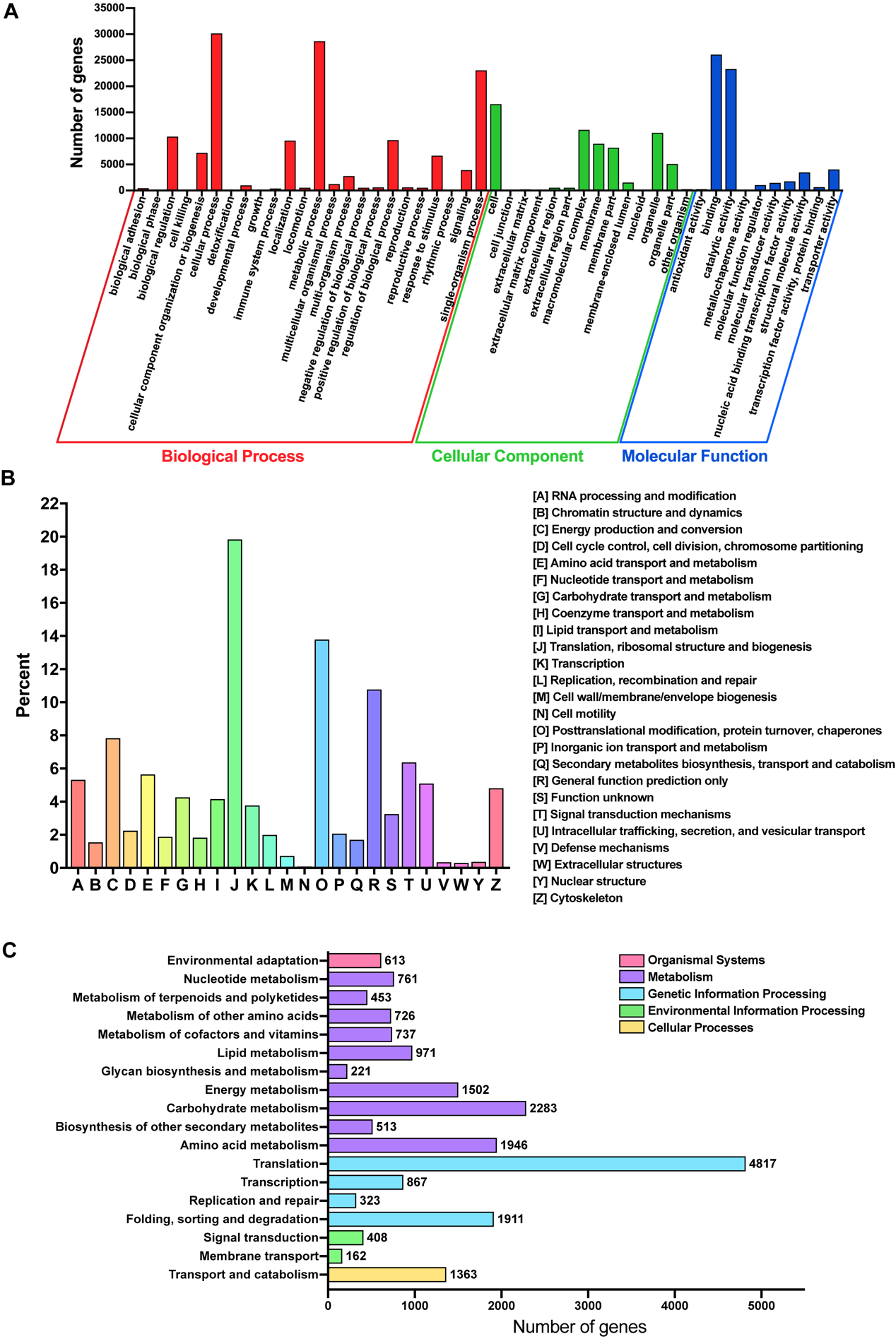
**

**Supplementary Figure S2** GO (A), COG (B) and KEGG (C) enrichment of assembled unigenes in *S. tetrandra*.

**
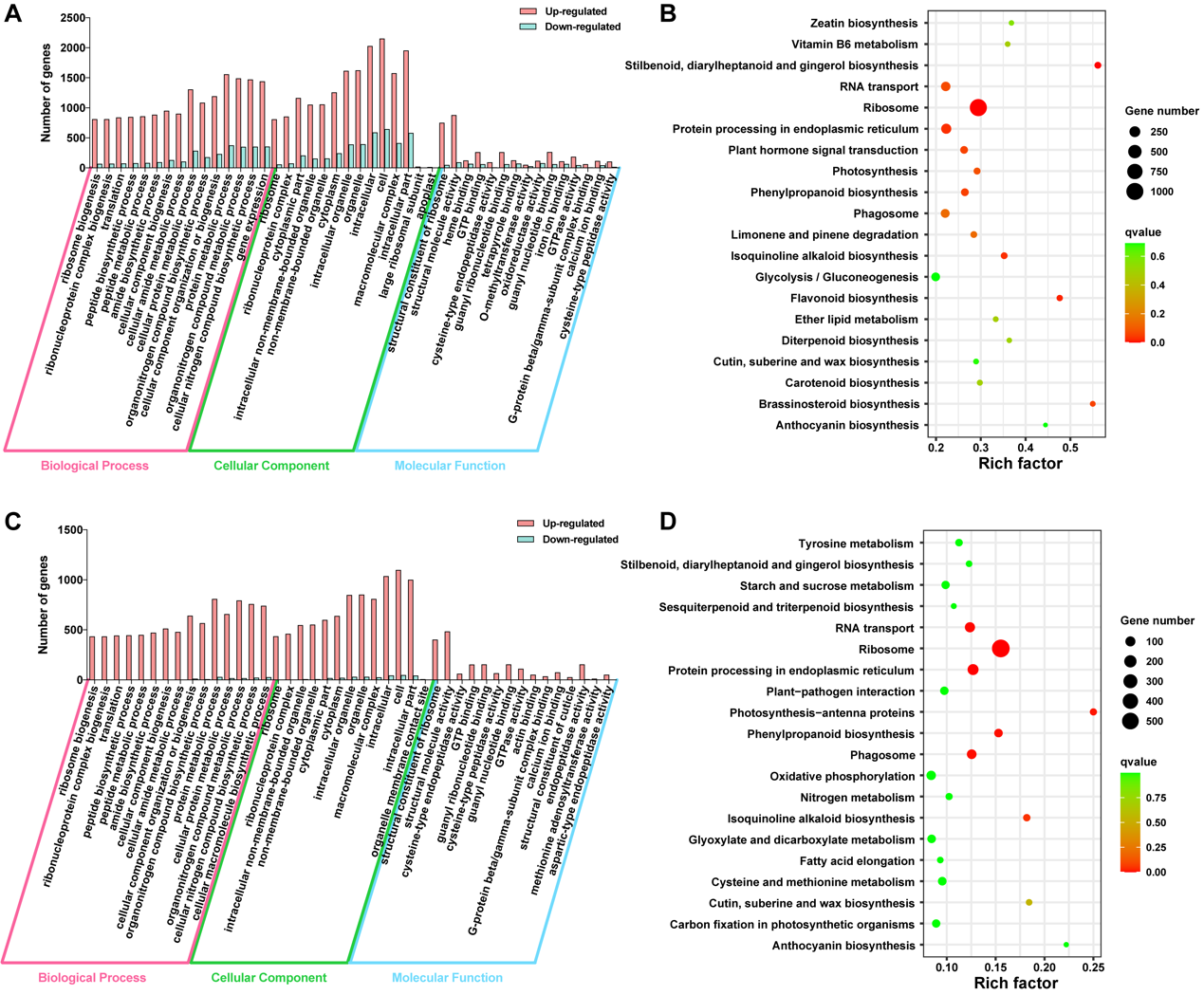
**

**Supplementary Figure S3** GO enrichment analysis of DEGs in epidermis vs. stem (A) and epidermis vs. xylem (C). Scatterplot of KEGG pathway enrichment of DEGs in epidermis vs. stem (B) and epidermis vs. xylem (D).

**
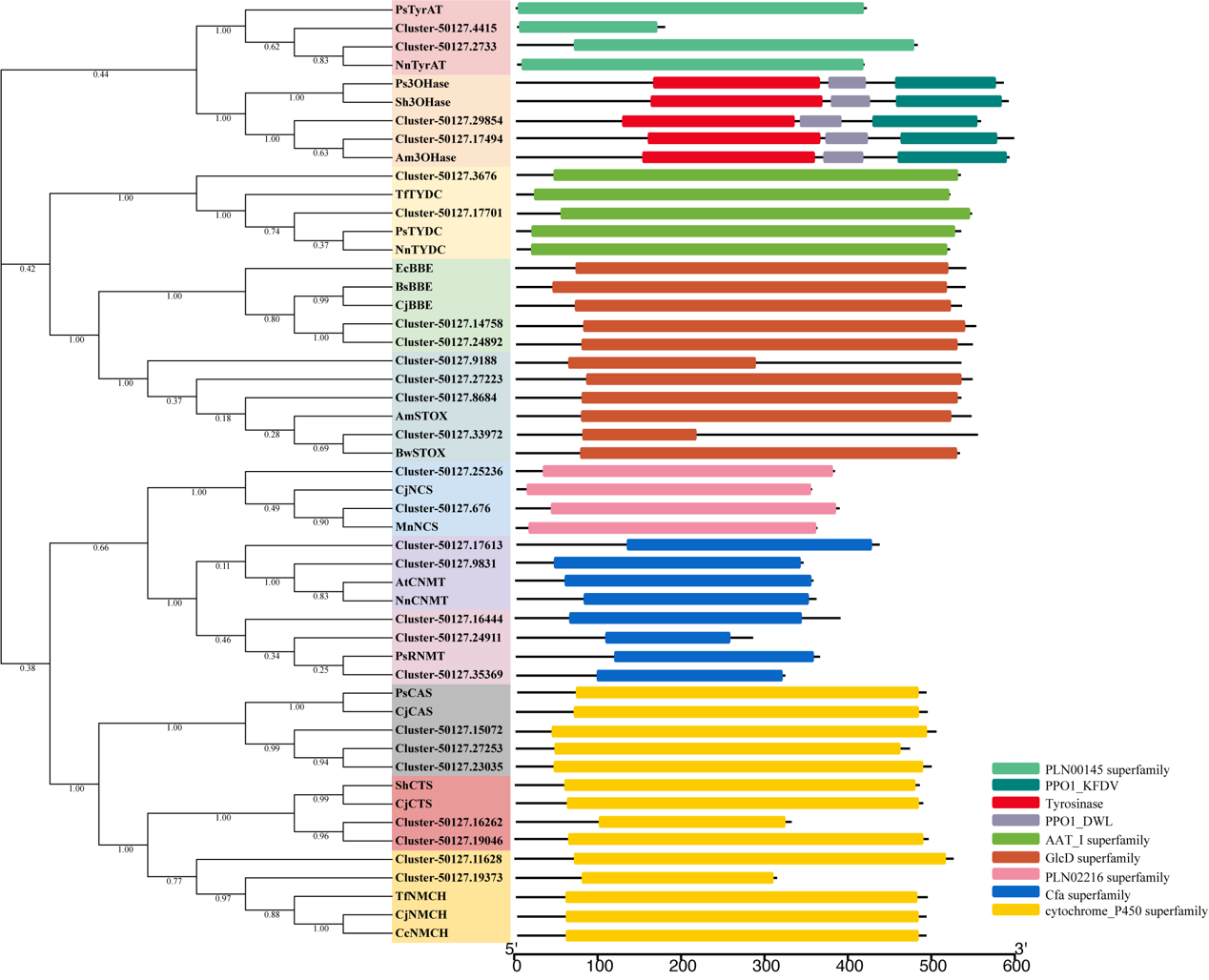
 Supplementary Figure S4** Phylogenetic tree and conserved motif structure of transcripts encoding enzymes related to BIA biosynthesis in *S. tetrandra* and reported enzymes from other plants.


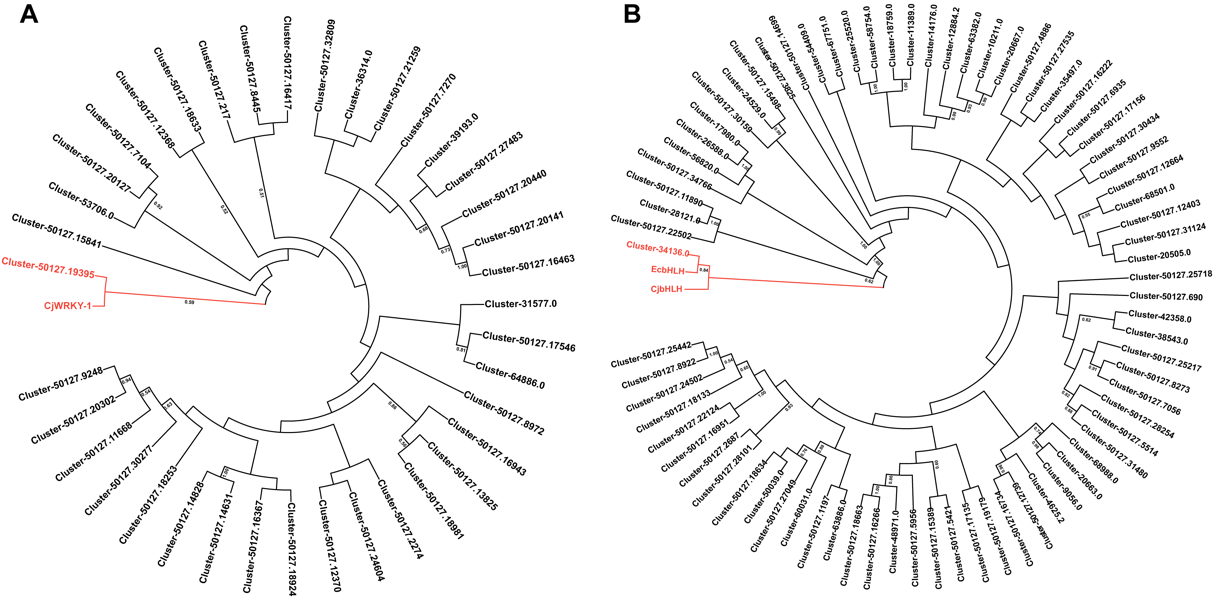


**Supplementary Figure S5** Phylogenetic tree of WRKY (A) and bHLH families (B) in *S. tetrandra* and reported proteins from other plants.


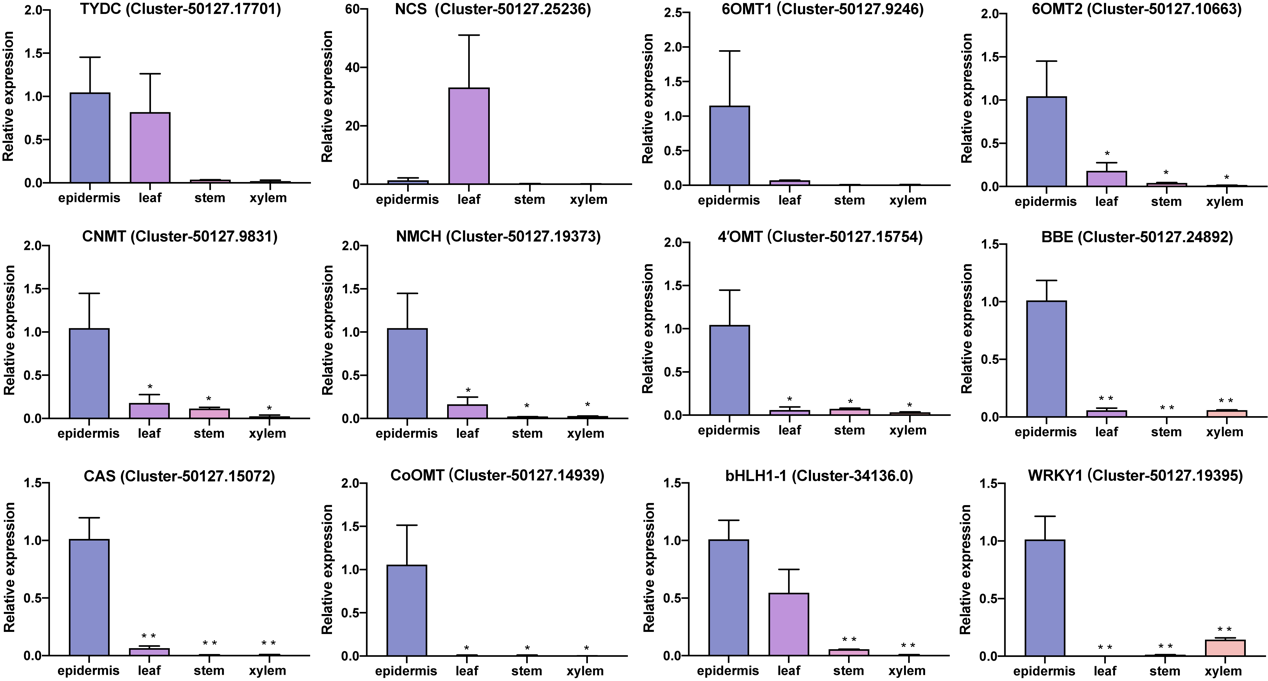


**Supplementary Figure S6** qRT-PCR analysis the expression levels of BIA biosynthesis-related genes. Actin was used as an internal control. Asterisks indicate statistically significant differences compared with epidermis lines: * P < 0.05, ** P < 0.01.

**
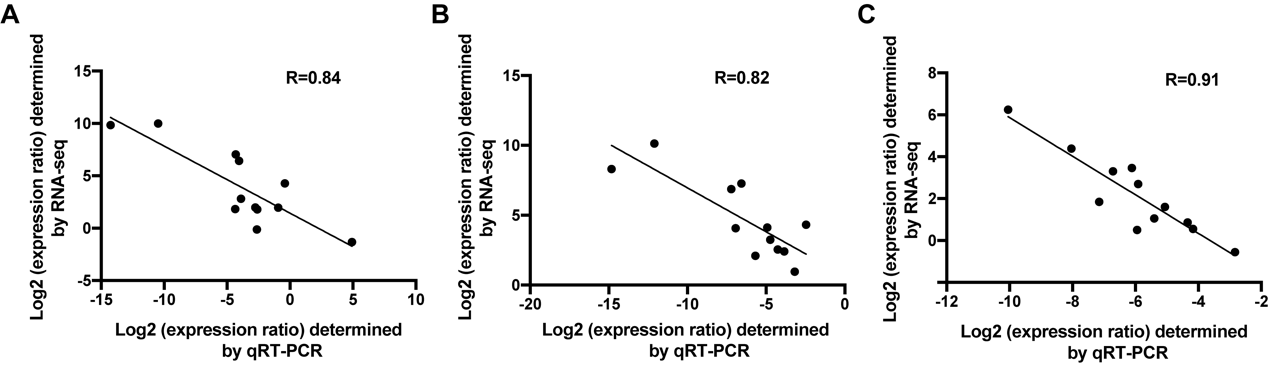
**

**Supplementary Figure S7** Correlation coefficients between qRT-PCR and RNA-Seq epidermis vs. leaf (A), epidermis vs. stem (B), and epidermis vs. xylem (C).

**
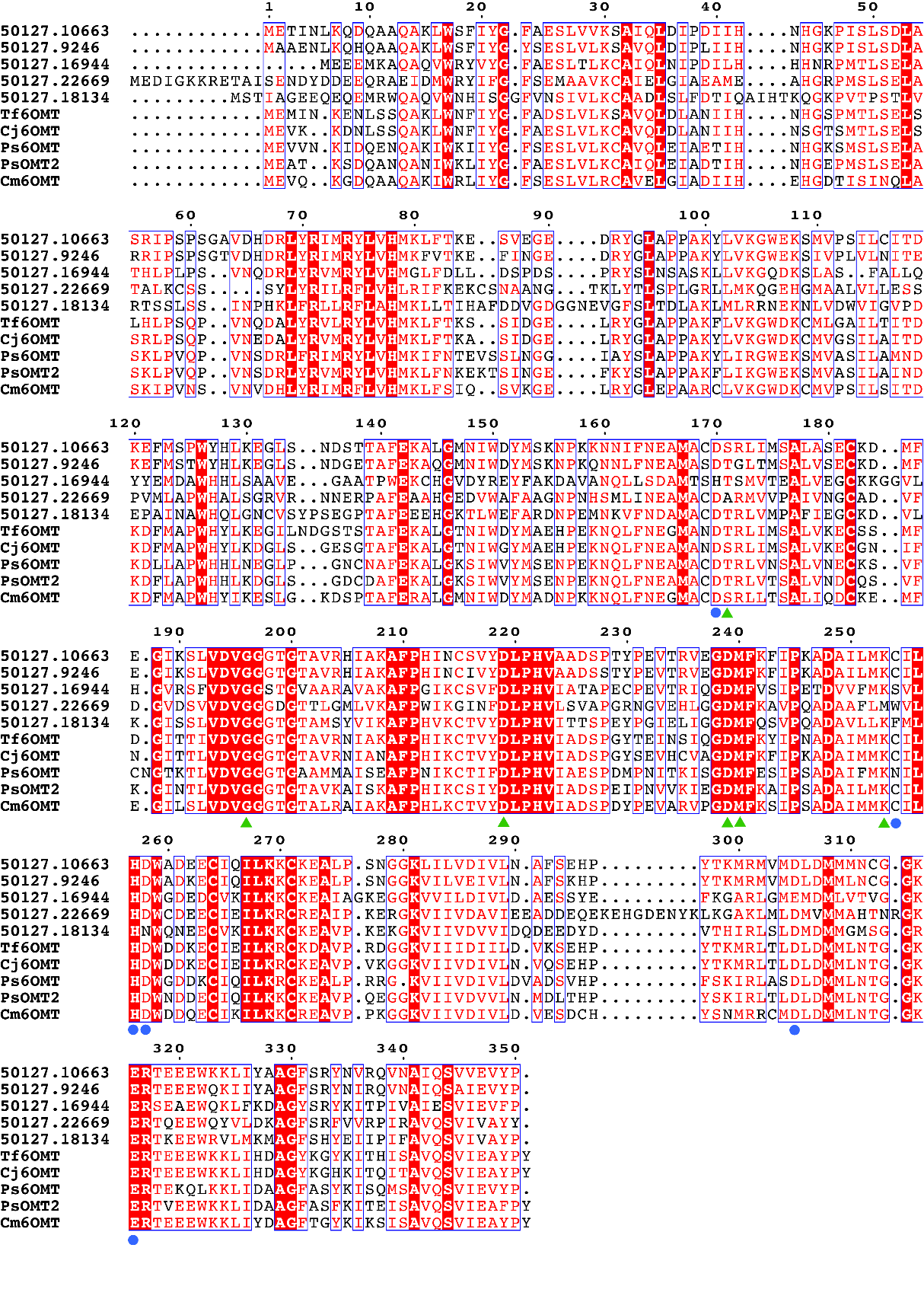
**

**Supplementary Figure S8** Multiple alignment of St6OMT2 with other 6OMTs. Numbering is based on the sequence of St6OMT2. Cyan closed cycles indicate the active sites of St6OMT2, and green triangles the SAM attachment sites of St6OMT2. The Cluster-50127.9246, Cluster-50127.16944, Cluster-50127.22669 and Cluster-50127.18134 were named St6OMT1, St6OMT2, St6OMT3 and St6OMT4 by Li et al., respectively.

**
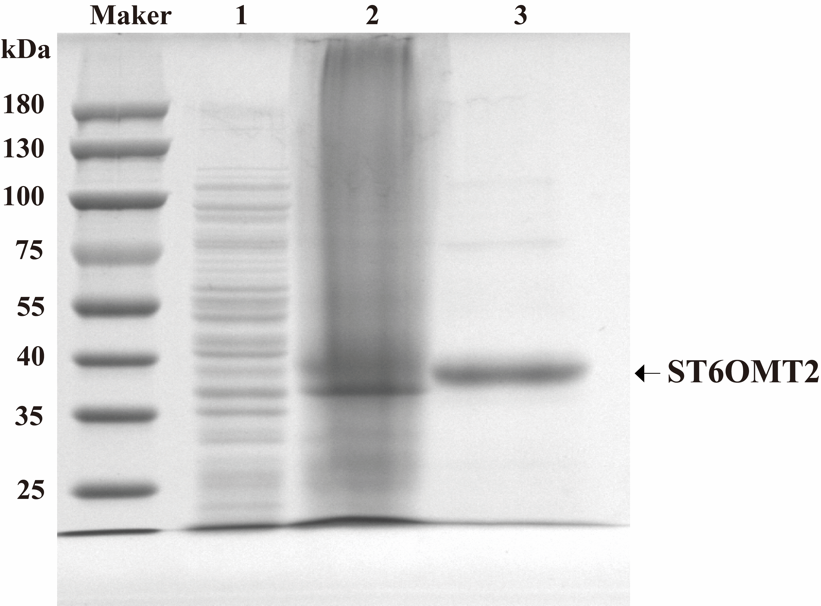
**

**Supplementary Figure S9** SDS-PAGE analyses of St6OMT2 expression in BL21 (DE3). Lane M: molecular weight protein marker; Lane 1: empty vector; Lane 2: supernatant of induced cell lysate; Lane 3: purified enzyme.

**Supplementary Table S1** Protein sequences information for the phylogenetic tree.

| **Enzyme abbreviation** | **Accession ID** | **Species** |
| --- | --- | --- |
| Am3OHase | ACJ76786.1 | *Argemone mexicana* |
| AmSTOX | ADY15027.1 | *A. mexicana* |
| AtCNMT | AAM65762.1 | *Arabidopsis thaliana* |
| BsBBE | AAD17487.1 | *Berberis stolonifera* |
| BwSTOX | ADY15026.1 | *B. wilsoniae* |
| CcNMCH | ABS19627.1 | *Coptis chinensis* |
| Cj4'OMT | Q9LEL5.1 | *C. japonica* |
| Cj6OMT | Q9LEL6.1 | *C. japonica* |
| CjBBE | BAM44344.1 | *C. japonica* |
| CjbHLH | BAJ40865.1 | *C. japonica* |
| CjCAS | BAB68769.1 | *C. japonica* |
| CjCoOMT | Q8H9A8.1 | *C. japonica* |
| CjCTS | BAF80448.1 | *C. japonica* |
| CjNCS | BAF45337.1 | *C. japonica* |
| CjNMCH | BAB12433.1 | *C. japonica* |
| CjWRKY-1 | BAF41990.1 | *C. japonica* |
| Cm6OMT | RWR73070.1 | *Cinnamomum micranthum f. kanehirae* |
| Ec6OMT | BAM37634.1 | *Eschscholzia californica* |
| Ec7OMT | BAE79723.1 | *E. californica* |
| EcBBE | AAC39358.1 | *E. californica* |
| EcbHLH | BAO74174.1 | *E. californica* |
| EgCaOMT1 | P46484.1 | *Eucalyptus gunnii* |
| Gel4OMT | Q84KK6.1 | *Glycyrrhiza echinata* |
| LjI4OMT | Q84KK4.1 | *Lotus japonicus* |
| MnNCS | XP_024026516.1 | *Morus notabilis* |
| Nn6OMT | AXJ91465.1 | *Nelumbo nucifera* |
| NnCNMT | AXJ91467.1 | *N. nucifera* |
| NnTYDC | XP_010245171.1 | *N. nucifera* |
| NnTyrAT | XP_010250299.1 | *N. nucifera* |
| PaCaOMT | XP_034899802.1 | *Populus alba* |
| Ps3OHase | XP_026389945.1 | *Papaver somniferum* |
| Ps4'OMT1 | Q7XB11.1 | *P. somniferum* |
| Ps4'OMT2 | Q7XB10.1 | *P. somniferum* |
| Ps6OMT | Q6WUC1.1 | *P. somniferum* |
| PsOMT2 | AKO60153.1 | *P. somniferum* |
| Ps7OMT | Q6WUC2.1 | *P. somniferum* |
| PsCAS | I3QBP4.1 | *P. somniferum* |
| PsRNMT | A0A1C9U5X5.1 | *P. somniferum* |
| PsTYDC | AAA62347.1 | *P. somniferum* |
| PsTyrAT | ADC33123.1 | *P. somniferum* |
| Sh3OHase | ALG05139.1 | *Sinopodophyllum hexandrum* |
| Sh6OMT | AJD20222.1 | *S. hexandrum* |
| ShCTS | AJD20229.1 | *S. hexandrum* |
| Tf6OMT | Q5C9L7.1 | *Thalictrum flavum subsp. glaucum* |
| TfNMCH | AAU20767.1 | *T. flavum subsp. glaucum* |
| TfTYDC | AAG60665.1 | *T. flavum subsp. glaucum* |
| Tt6OMT | KAF5202636.1 | *T. thalictroides* |
| TtCoOMT | KAF5182903.1 | *T. thalictroides* |

**Supplementary Table S2** Primers used in this study.

| **Name** | **Sequence (5' to 3')** |
| --- | --- |
| 6OMT2-F | GCAAATGGGTCGCGGATCCATGGAAACAATTAATCTAAAGCAAGAC |
| 6OMT2-R | CGAGTGCGGCCGCAAGCTTTCAAGGGTAAACTTCAACAACAGAC |
| qPCR-Actin-F | AACAAATTGGACGCAGTAGCA |
| qPCR-Actin-R | GCTTAGCACTTTCCAGCAGAT |
| qPCR-TYDC-F | AAGCCACTGAATCTAAGCAGGTTGT |
| qPCR-TYDC-R | ATTCGCCACTCCGTAGCTCCTC |
| qPCR-NCS-F | AGCATTGAACACAGAGGCACCAT |
| qPCR-NCS-R | AACAGTCTTGTAATTGGCACCAGTG |
| qPCR-6OMT1-F | CGAAAGACTCTGTTGAAGGCGAAG |
| qPCR-6OMT1-R | GAAGGCGGTAGTGCTGTCGTT |
| qPCR-6OMT2-F | CTGTCTACGATCTCCCTCATGTTGT |
| qPCR-6OMT2-R | GGATGGCATCAGCACTAGGAATGT |
| qPCR-CNMT-F | ACTATGCCTTCTGCGAACCTATTGT |
| qPCR-CNMT-R | GGTCCATTCTCTTCAACCACTCTTC |
| qPCR-NMCH-F | GGGCATTGGTGAACATCTTTGGTAG |
| qPCR-NMCH-R | AAGTAATCAGCAGGATTGGCAGAGT |
| qPCR-4′OMT-F | ATGACAGTGTTGGACTTGGCTTC |
| qPCR-4′OMT-R | CTGGTTGGATTGAGTAACGCTTGAG |
| qPCR-BBE-F | GCCGAAACTCCCTTTGTCCTCATAG |
| qPCR-BBE-R | TCGCTTGCCTGACTGATTGTGTAAT |
| qPCR-CAS-F | GTCGTCAAGCCTCTTAACCATCTCC |
| qPCR-CAS-R | CGTCCTCGATCAGCTCATCCATTC |
| qPCR-CoOMT-F | CATAACGCTGACCGAACTGGCTAG |
| qPCR-CoOMT-R | CTCTCCGCTCACTTCATCTGTTGT |
| qPCR-bHLH-F | GCGGAGGAAGTCAATGCGAGAG |
| qPCR-bHLH-R | CGGCGATGGGAATGTACTTGATGAT |
| qPCR-WRKY-F | CAACGGTAGGCGATGAGGTGATG |
| qPCR-WRKY-R | TCAACTTGGCTTCTTGTCTGAAACG |

**Supplementary Table S3** The relative content analysis of 12 BIAs by UPLC-Q-TOF/MS in *S. tetrandra.*

| **Composition** | **Molecular** | **[M+H]** | **Rt (min)** | **Average peak area** | | | |
| --- | --- | --- | --- | --- | --- | --- | --- |
|  |  |  |  | **Epidermis** | **Stem** | **Xylem** | **Leaf** |
| (S)-Norcoclaurine | C_16_H_17_NO_3_ | 272.1325 | 3.15 | 108303 | 2685 | 36888 | 4159 |
| (S)-Coclaurine | C_17_H_19_NO_3_ | 286.1425 | 5.43 | 99195 | 11188 | 54760 | 32607 |
| (S)-N-Methylcoclaurine | C_18_H_21_NO_3_ | 300.1638 | 5.26 | 4431 | 2525 | 4407 | ND |
| Fangchinoline | C_37_H_40_N_2_O_6_ | 609.2988 | 14.26 | 1640854 | 26775 | 4060765 | 63678 |
| Tetrandrine | C_38_H_42_N_2_O_6_ | 623.3116 | 17.26 | 6110144 | 55479 | 5487276 | 109543 |
| (S)-Reticuline | C_19_H_23_NO_4_ | 330.1714 | 6.72 | 4477 | 3089 | 4272 | ND |
| (S)-Corytuberine | C_19_H_21_NO_4_ | 328.1549 | 6.83 | ND | ND | 194487 | ND |
| Magnoflorine | C_20_H_24_NO_4_^+^ | 343.1793 | 8.63 | 1646556 | ND | 1870833 | ND |
| (S)-Scoulerine | C_19_H_21_NO_4_ | 328.1549 | 3.40 | ND | ND | 194487 | ND |
| (S)-Tetrahydrocolumbamine | C_20_H_23_NO_4_ | 342.1705 | 8.61 | 4445884 | 855164 | 1171331 | 97544 |
| Tetrahydropalmatine | C_21_H_25_NO_4_ | 356.1862 | 12.55 | 621975 | ND | 605801 | ND |
| Berberine | C_20_H_18_NO_4_^+^ | 337.1314 | 9.62 | 72346 | 72774 | 72337 | 68108 |

Rt: Retention time; ND: No detected.

**Supplementary Table S4** Standard curve and sample detection of Fangchinoline and Tetrandrine.

|  | Fangchinoline | Tetrandrine |
| --- | --- | --- |
| Regression equation | y = 23.682x + 14.232 | y = 25.428x + 43.588 |
| Correlation coefficient (r) | 0.9959 | 0.9973 |
| Linear range (µg mL^-1^) | 0.156-10 | 1.25-10 |
| Content in epidermis (mg g^-1^) | 8.37±0.37 | 17.31±0.69 |
| Content in xylem (mg g^-1^) | 11.47±1.56 | 8.39±0.60 |
| Content in stem (mg g^-1^) | BLQ | BLQ |
| Content in leaf (mg g^-1^) | ND | ND |

BLQ, Beneath Limit of Quantification; ND, None Detected

**Supplementary Table S5** Summary of the Illumina data.

| **Sample** | **Raw reads** | **Clean reads** | **Clean bases** | **Error rate** | **Q20** | **Q30** | **GC percent (%)** |
| --- | --- | --- | --- | --- | --- | --- | --- |
| E1 | 59493878 | 57209390 | 8.58 G | 0.02 | 98.2 | 94.6 | 46.48 |
| E2 | 61066480 | 58610682 | 8.79 G | 0.02 | 98.2 | 94.8 | 46.51 |
| E3 | 59277660 | 56454976 | 8.47 G | 0.02 | 98.2 | 94.6 | 46.32 |
| L1 | 66503196 | 59174088 | 8.88 G | 0.02 | 98.2 | 94.6 | 44.89 |
| L2 | 62133136 | 59619420 | 8.94 G | 0.02 | 98.2 | 94.7 | 45.18 |
| L3 | 61503656 | 59630964 | 8.94 G | 0.02 | 98.1 | 94.4 | 44.53 |
| S1 | 61709208 | 58808084 | 8.82 G | 0.02 | 98.2 | 94.5 | 45.2 |
| S2 | 43869172 | 43379292 | 6.51 G | 0.03 | 97.7 | 93.2 | 45.68 |
| S3 | 68851844 | 64983316 | 9.75 G | 0.02 | 98.1 | 94.6 | 45.25 |
| X1 | 59087992 | 57634024 | 8.65 G | 0.02 | 98.1 | 94.4 | 46.19 |
| X2 | 70822138 | 67249920 | 10.09 G | 0.02 | 98.3 | 94.8 | 46.32 |
| X3 | 49303424 | 46782152 | 7.02 G | 0.02 | 98.3 | 94.8 | 46.35 |

E: epidermis; L: leaf; S: stem; X: xylem.

**Supplementary Table S6** KEGG pathways for biosynthesis of other secondary metabolites annotated in *S. tetrandra*.

| **KEGG Pathway** | **Pathway ID** | **Number of Unigenes** |
| --- | --- | --- |
| Anthocyanin biosynthesis | ko00942 | 9 |
| Betalain biosynthesis | ko00965 | 6 |
| Caffeine metabolism | ko00232 | 28 |
| Carbapenem biosynthesis | ko00332 | 3 |
| Flavone and flavonol biosynthesis | ko00944 | 12 |
| Flavonoid biosynthesis | ko00941 | 43 |
| Glucosinolate biosynthesis | ko00966 | 13 |
| Indole alkaloid biosynthesis | ko00901 | 3 |
| Isoflavonoid biosynthesis | ko00943 | 7 |
| Isoquinoline alkaloid biosynthesis | ko00950 | 88 |
| Monobactam biosynthesis | ko00261 | 51 |
| Phenylpropanoid biosynthesis | ko00940 | 242 |
| Stilbenoid, diarylheptanoid and gingerol biosynthesis | ko00945 | 57 |
| Tropane, piperidine and pyridine alkaloid biosynthesis | ko00960 | 77 |

**Supplementary Table S7** The enzymatic properties of 6OMT have been reported.

| **Protein** | **Plant species** | **Substrates** | **pH** | **Temperature (℃)** | ***K_m_* (μΜ)** | ***k_cat_* (S^-1^)** | **Reference** |
| --- | --- | --- | --- | --- | --- | --- | --- |
| ST6OMT2 | *Stephania tetrandra* | norcoclaurine | 6.0 | 30 | 28.9±10 | 1.5±0.1 | This study |
| GflOMT2 | *Glaucium flavum* | norlaudanosoline | ND | ND | 18.3±2.5 | 2.78±0.09 | (Chang et al., 2015) |
|  |  | scoulerine | ND | ND | 31.0±5.0 | 1.25±0.06 |  |
| NnOMT1 | *Nelumbo nucifera* | (R,S)-norcoclaurine | 8.0 | 30 | 20±2 | 0.0336 | (Menéndez-Perdomo and Facchini, 2020) |
| Cj6OMT | *Coptis japonica* | (R,S)-norlaudanosoline | 9.0 | ND | 2200 | ND | (Morishige et al., 2000) |
|  |  | norreticuline | ND | ND | 103 | ND |  |
| PsOMT2 | *Papaver somniferum* | (R,S)-norcoclaurine | 6.0-9.0 | 37-41 | 10 | ND | (Ounaroon et al., 2003) |
|  |  | (R)-norprotosinomenine | 7.5 | 37-41 | 5 | ND |  |
|  |  | (S)-norprotosinomenine | 7.5 | 37-41 | 5 | ND |  |
|  |  | (R,S)-isoorientaline | 7.5 | 37-41 | 29 | ND |  |
| Tf6OMT | *Thalictrum flavum subsp. glaucum* | (R,S)-norlaudanosoline | ND | ND | 15±2 | 0.15 | (Robin et al., 2016) |

**Reference**

Chang, L., Hagel, J. M., and Facchini, P. J. (2015). Isolation and characterization of O-methyltransferases involved in the biosynthesis of glaucine in *Glaucium flavum*. Plant Physiol*.* 169, 1127–1140. doi:10.1104/pp.15.01240.

Menéndez-Perdomo, I. M., and Facchini, P. J. (2020). Isolation and characterization of two O-methyltransferases involved in benzylisoquinoline alkaloid biosynthesis in sacred lotus (*Nelumbo nucifera*). J. Biol. Chem*.* 295, 1598–1612. doi:10.1074/jbc.RA119.011547.

Morishige, T., Tsujita, T., Yamada, Y., and Sato, F. (2000). Molecular characterization of the S-adenosyl-L-methionine:3’-hydroxy-N-methylcoclaurine 4’-O-methyltransferase involved in isoquinoline alkaloid biosynthesisin *Coptis japonica*. J. Biol. Chem*.* 275, 23398–23405. doi:10.1074/jbc.M002439200.

Ounaroon, A., Decker, G., Schmidt, J., Lottspeich, F., and Kutchan, T. M. (2003). (R,S)-Reticuline 7-O-methyltransferase and (R,S)-norcoclaurine 6-O-methyltransferase of *Papaver somniferum* - cDNA cloning and characterization of methyl transfer enzymes of alkaloid biosynthesis in *opium poppy*. Plant J*.* 36, 808–819. doi:10.1046/j.1365-313X.2003.01928.x.

Robin, A. Y., Giustini, C., Graindorge, M., Matringe, M., and Dumas, R. (2016). Crystal structure of norcoclaurine-6-O-methyltransferase, a key rate-limiting step in the synthesis of benzylisoquinoline alkaloids. Plant J*.* 87, 641–653. doi:10.1111/tpj.13225.
